# Middle Pleistocene Hominin Teeth from Longtan Cave, Hexian, China

**DOI:** 10.1371/journal.pone.0114265

**Published:** 2014-12-31

**Authors:** Song Xing, María Martinón-Torres, José María Bermúdez de Castro, Yingqi Zhang, Xiaoxiao Fan, Longting Zheng, Wanbo Huang, Wu Liu

**Affiliations:** 1 Key Laboratory of Vertebrate Evolution and Human Origins of Chinese Academy of Sciences, Institute of Vertebrate Paleontology and Paleoanthropology, Chinese Academy of Sciences, Beijing, China; 2 National Research Center on Human Evolution (CENIEH), Burgos, Spain; 3 Hexian Museum of Anhui Province, Hexian, China; 4 Anhui Museum, Hefei, China; 5 Chongqing Three Gorges Institute of Paleoanthropology, China Three Gorges Museum, Chongqing, China; University of Delaware, United States of America

## Abstract

Excavations at the Longtan Cave, Hexian, Anhui Province of Eastern China, have yielded several hominin fossils including crania, mandibular fragments, and teeth currently dated to 412±25 ka. While previous studies have focused on the cranial remains, there are no detailed analyses of the dental evidence. In this study, we provide metric and morphological descriptions and comparisons of ten teeth recovered from Hexian, including microcomputed tomography analyses. Our results indicate that the Hexian teeth are metrically and morphologically primitive and overlap with *H. ergaster* and East Asian Early and mid-Middle Pleistocene hominins in their large dimensions and occlusal complexities. However, the Hexian teeth differ from *H. ergaster* in features such as conspicuous vertical grooves on the labial/buccal surfaces of the central incisor and the upper premolar, the crown outline shapes of upper and lower molars and the numbers, shapes, and divergences of the roots. Despite their close geological ages, the Hexian teeth are also more primitive than Zhoukoudian specimens, and resemble Sangiran Early Pleistocene teeth. In addition, no typical Neanderthal features have been identified in the Hexian sample. Our study highlights the metrical and morphological primitive status of the Hexian sample in comparison to contemporaneous or even earlier populations of Asia. Based on this finding, we suggest that the primitive-derived gradients of the Asian hominins cannot be satisfactorily fitted along a chronological sequence, suggesting complex evolutionary scenarios with the coexistence and/or survival of different lineages in Eurasia. Hexian could represent the persistence in time of a *H. erectus* group that would have retained primitive features that were lost in other Asian populations such as Zhoukoudian or Panxian Dadong. Our study expands the metrical and morphological variations known for the East Asian hominins before the mid-Middle Pleistocene and warns about the possibility that the Asian hominin variability may have been taxonomically oversimplified.

## Introduction

During the decades of the 1970s and the 1980s, surveys and excavations at the Longtan Cave, Anhui County, Eastern China, uncovered a nearly complete skullcap, a left partial mandibular corpus with two teeth in situ, and ten isolated teeth among some other fragmentary hominin fossils [1]. Since their discovery, most of the studies have focused in the cranial elements [2]–[10] pointing to the expression of features that are classically considered typical of Asian *H. erectus*. Although there was no great dispute regarding its attribution to *H. erectus*, divergent views emerged on the primitive or derived status of the Hexian sample with regard to the Zhoukoudian or the Indonesian hypodigms. These studies portrayed the high morphological variability of the Asian human fossil record and how far we were from an interpretative consensus over its evolutionary meaning. In addition, the scientific debate has primarily focused on the cranial remains, paying little attention to the abundant dental evidence. Because variation in dental form is highly heritable, paleoanthropologists consider that teeth may be more useful than other skeletal elements for assessing the affinities of extant and extinct human populations [11]–[14] and thus, to assess the taxonomic and phylogenetic position of a sample. Only recently, new detailed and specialized dental studies of old and recent Asian discoveries [15]–[21] have allowed a more precise characterization of these hominins, contributing with new evidence to the understanding of human evolution in Asia.

For the past three decades, several Early and Middle Pleistocene hominin fossils have been found at many sites in Africa, Asia, and Europe [1], [22]–[26]. This increasing body of evidence becomes a crucial piece in understanding the variability of the human fossil record. Here, we present the first detailed comparative study of the Hexian dental evidence including the application of microcomputed tomography (micro-CT). The morphological and metric comparison of this sample will be framed in the discussion about what is known at present about the variability of the Asian fossil record.

## The Hexian Site

**Figure 1:**
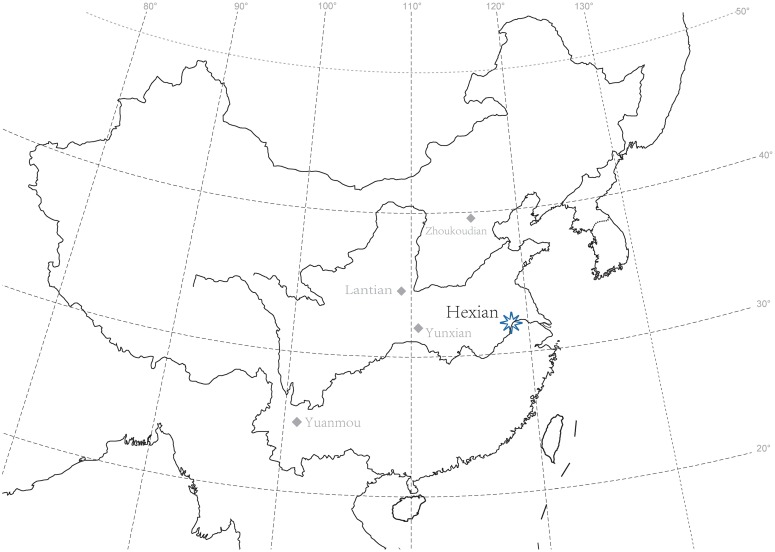
Geographic location of the Longtan Cave, Hexian.

Longtan Cave (Longtandong) is located at Hexian County, Anhui Province, China, just to the north of the Yangtze River (31°45′N, 118°20′E) (Fig. 1). During the decades of the 1970s and 1980s, surveys and excavations at the cave uncovered several faunal remains. In July, 1980, a human left upper second molar was found during a survey. During 1980 and 1981, three excavations were conducted at Hexian, and in these excavations, more hominin fossils were unearthed.

**Figure 2:**
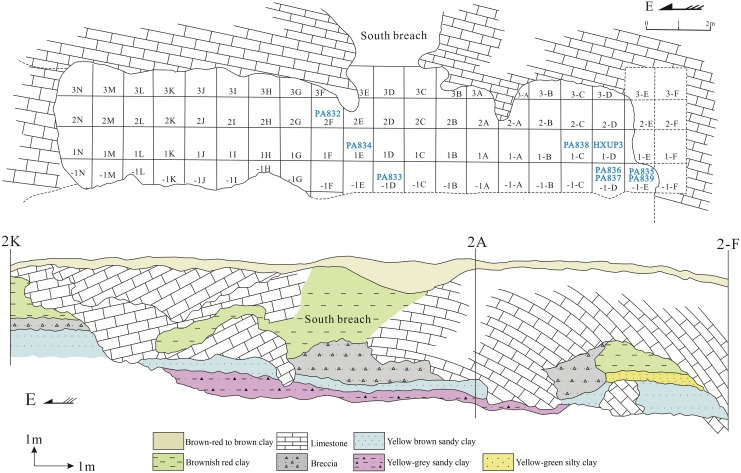
Plan view of the Longtan Cave excavation area (upper) and stratigraphic layers (lower) (modified after Yongxiang Ye).

Fig. 2 shows the plan view of the Longtan Cave excavation area and the stratigraphic profile. According to Huang et al. [3], five clear stratigraphic layers can be identified at the Longtan Cave (Fig. 2). From bottom to top the layers are described as follows:

- Layer 1: a yellow-grey sandy clay, approximately 1.5 m thick;
- Layer 2: a yellow-brown sandy clay of variable thickness (0.7 to 1.4 m) containing the human remains and nearly all other faunal material;
- Layer 3: a yellow-green silty clay of 0.1 to 0.3 m thickness;
- Layer 4: a brownish red clay, approximately 2.3 m thick;
- Layer 5: a brown-red to brown clay of 0.2 to 0.4 m thickness.

The hominin fossils found at the Longtan Cave comprise a nearly complete skullcap, two cranial fragments, a fragmentary left mandibular corpus with the M_2_ and the M_3_ in situ, and ten isolated teeth. The location of each tooth at the excavation area is marked in Fig. 2. Faunal assemblage comprises remains from more than fifty mammal species, showing a mixture of cold adapted northern mammals and more subtropical-tropical southern elements [1]. No artifacts were found at the site, but the hominin activities have been confirmed through the cut mark analysis of the surface of the mammal fossils and their pattern of fragmentation [1], [3].

Different methods have been used to date the Hexian hominin fossils (see S1 Table in S1 Text for summary). According to Huang et al. [2], the Hexian fauna has three main components. First, we can identify genera such as *Stegodon*, *Tapirus*, and *Megatapirus*, which belong to the *Ailuropoda-Stegodon* fauna commonly seen in South China. Second, there are some typical components of the Zhoukoudian faunal assemblage, e.g. *Sinomegaceros pachyosteus*, *Hyaena sinensis*, *Trogontherim*, and *Pseudaxis grayi.* Third, there are a few members that are typical from East China. Thus, the faunal combination at Hexian indicates a mixed and transitional scenario between North and South China. The archaic or Early Pleistocene faunal components are missing. In addition, the advanced dental morphologies of *Megantereon sp*. and *Trogontherium cuvieri Fischer*, as well as the less robust mandible of *Megaloceros pachyosteus* matches the deposits found at the fifth layer of Zhoukoudian Locality 1 and thus, it would correspond to approximately the mid-Middle Pleistocene period [27].

Pollen analysis [27] point to an uplift of the Longtan Cave during the transition between layers 3 and 4, which match well the active crustal movement that happened during the Middle and Late Pleistocene.

Initial dating analysis, usually based on ESR or U-series [28]–[31], indicated that Hexian hominins might have lived in the same period as the late members of Zhoukoudian Locality 1. However, Grün et al. [32] criticized the reliability of the stand-alone ESR or U-series age results due to the difficulties to estimate the U-uptake history of the teeth. For this reason, we rely in the estimations of Grün et al., [32] as they are based on a combined ESR and U-series method [32]. The interval of 412±25 ka they obtain for the Hexian hominins is also consistent with the faunal composition.

Faunal taphonomy and pollen analysis suggest a rapid deposition of the Layer 2 that contains the hominin fossils and most faunal remains [3], [27], [32]. These studies indicated clear climate boundaries among the five depositional layers, although all the Hexian deposits (Layer 2) formed in the same sub-tropical period before the uplift of the Longtan Cave. In addition, the taphonomical analysis of the accumulation in Hexian Layer 2 provides no evidence of secondary re-deposition. In those places with a higher fauna accumulation, the abundant postcranial bones of rhinoceros, bovine, and cervidae are piled without a preferential orientation of their long axis (see [27] for further details). In addition, cut marks on mammal fossils, as well as tools made from bone, antler, and teeth were also found [27]. In the west side of the cave, there were also evidences of burned bones and teeth [27]. This depositional context suggests that Longtan Cave could have been a living place for Hexian hominins and that the deposit of Layer 2 is original. From all these we can assume that, in geological terms, all the human fossils were accumulated in a relatively short period and have the same geo-chronological age.

## Materials and Methods

### Materials

In this study, ten isolated hominin teeth from the Longtan Cave at Hexian are described and analyzed (Table 1). Observations were made on originals except for the upper central incisor, where a high resolution cast was used. Apart from these ten teeth, there are two molars attached to a mandibular fragment that have not been included in our analysis because they are not available for study.

**Table 1:** Hominin teeth from the Longtan Cave of Hexian used for present study (Measurements in brackets are estimations due to the severe occlusal and interproximal wear).

Specimen No.	Tooth type	Side	Crown measurements (mm)		Museum collections
			MD	BL	
PA832	Upper third premolar (P^3^)	right	9.0	13.4	IVPP
HXUP3	Upper third premolar (P^3^)	left	9.3	13.2	Hexian Museum
PA836	Upper first molar (M^1^)	left	12.3	13.7	IVPP
PA833	Upper second molar (M^2^)	left	12.0	14.0	IVPP
PA837	Upper second molar (M^2^)	right	12.5	15.5	IVPP
PA838	Lower second molar (M_2_)	left	13.6	13.9	Anhui Museum
PA839	Lower second molar (M_2_)	left	14.3	13.4	IVPP
PA834-1	Lower second molar (M_2_)	left	(12.5)	(13.1)	IVPP
PA834-2	Lower third molar (M_3_)	left	13.6	13.6	IVPP
PA835	Upper central incisor (I^1^)	right	11.7	9.4	IVPP

The Hexian hominins are mainly compared with those from the Early and Middle Pleistocene of Africa, Asia, and Europe. In order to explore the polarity of the observed morphologies, samples of *Australopithecus*, African early *Homo*, and *H. sapiens* are also included (Table 2 and S2 Table in S1 Text). For the morphological study, the recent *H. sapiens* specimens from Asia were sourced from Henan and Hubei Province, Central China, with an archaeological time span from Neolithic to Qing Dynasty. The modern human samples from Africa include several Mesolithic collections and the samples from Europe range from the Neolithic to medieval times. The crown size data of recent *H. sapiens* was taken from Frayer’s work [33] (S3 Table in S1 Text).

**Table 2:** Specimens used in the morphological and metric comparisons.

Geography and Chronology	Specimens
**Africa**	
Pliocene (*Australopithecus*)	Hadar, Laetoli, Makapansgat*, Sterkfontein*
Late Pliocene and Early Pleistocene (Early *Homo*)	East Rudolf*, Olduvai, Sterkfontein*
Late Pliocene and Early Pleistocene (*H. ergaster*)	East Rudolf*, Olduvai, Sterkfontein*, West Turkana*
North African Middle Pleistocene Holocene (Recent modern human)	Rabat, Sidi Abderrhaman, Ternifine*, Thomas Quarry Mesolithic North African Sample (Tebessa, Aïn Meterchem, Gambetta, Aïn Dokkara, Taforalt)*
**East Asia**	
Early Pleistocene	Jianshi*, Sangiran*, Yuanmou
Mid-Middle Pleistocene	Chenjiawo*, Zhoukoudian (ZKD) Locality 1
Late Middle Pleistocene	Chaoxian*, Panxian Dadong*
Late Pleistocene (Early modern humans)	Huanglong Cave*, Liujiang*, Xintai* Zhiren Cave*, Zhoukoudian (ZKD) Upper Cave
Holocene (Recent modern human)	Contemporary modern sample from China* (Henan Province and Hubei Province)
**West Asia**	
Early Pleistocene	Dmanisi*
Late Pleistocene (Early modern human)	Qafzeh*, Skhul
**Europe**	
Early Pleistocene	Atapuerca Gran Dolina (TD6)*
Middle Pleistocene	Atapuerca Sima de los Huesos (SH)*, Arago*, Mauer, Montmaurin*, Petralona, Steinheim
Neanderthals	Amud, Arcy-sur-Cure*, Breuil, Cabezo Gordo, Chateauneuf, Ehringsdorf, El Sidrón, Genay (Côte d’Or), Gibraltar, Guattari, Hortus*, Krapina, Kulna, La Chaise, La Ferrassie*, La Quina*, Monsempron*, Le Moustier, Ochoz, Pech de l’Azé, Petit-Puymoyen*, Pinilla del Valle*, Regourdou, Saccopastore*, Sakajia, Shanidar, Spy, St. Césaire, Subalyuk, Tabun*, Vindija, Zafarraya*
Late Pleistocene (Early modern human)	Abri Pataud*, Brassempouy, Combe Capelle, Dolní Vstonice*, Cro-Magnon, Fontechevade, Isturitz*, Grimaldi, Le Rois*, L’Espugo*, Les Vachons, Mladeč, Pavlov*, Predmostí, Saint Germain-La Riviere*, Trou Magrite, Zlaty Kun
Holocene (Recent modern human)	Upper Paleolithic and Mesolithic European samples (Denmark, France, Germany, Portugal, Sweden) [33]; Hispanic-muslim medieval collection of San Nicolás, Murcia*, Spain; Mesolithic French sample (Tévic and Hoëdic)*; Neotlithic French sample (Avize, Dolmens de Bretons, Caverne de L’Homme Mort, Orrouy)*

Note: “*” means that we examined the original fossil. For the rest, we employed high resolution casts.

Due to the uncertainty and dispute over the taxonomic classification of some of the fossils, the comparative samples were mainly grouped based on their geographic location and geological age with only two exceptions. Since there may be more than one *Homo* species represented in the East Rudolf and Olduvai samples we have made two different groups for the African Late Pliocene and Early Pleistocene fossils. We will use the term Early *Homo* to refer to more primitive forms usually attributed to *H. habilis* and/or *H. rudolfensis* [34]–[38]. For the more advanced forms we will use the term *H. ergaster* [36], [37], [39]. The second exception refers to Neanderthals which will be treated as a separate taxonomic group to simplify the nomenclature for the Upper Pleistocene fossils and because, on dental grounds, their uniqueness is generally well-recognized [14], [40]–[43].

Regarding the Sangiran material, the precise stratigraphic and chronological context of the fossils is still a matter of discussion. The Sangiran Dome consists of two main formations: the Pucangan (also referred as Sangiran) and the Kabuh (also referred as Bapang) Formations. The Pucangan Formation is usually referred as Early Pleistocene [44]–[48]. Regarding the Kabuh Formation, some authors defend an Early Pleistocene range (e.g., [47]) whereas other researchers support a late Early-early Middle Pleistocene chronology (e.g., [49], [50]). Despite the lack of consensus, we can generally agree that the Pucangan material is earlier than the Bapang material although a large overlap cannot be disregarded. According to Zanolli [20] this chronological sequence could explain certain trends in the morphological variability of the Indonesian fossils. Thus, although the Indonesian material is globally referred to as Early Pleistocene, in the discussion we will make some distinctions about the Pucangan and the Kabuh Formations if it is relevant for our interpretation.

## Methods

Tooth wear stages are determined according to Molnar [51]. The dental morphological descriptions and comparisons were conducted following the terminology terminologies employed in Weidenreich [52], Bermúdez de Castro [53], Scott and Turner [54], and Martinón-Torres et al. [55]. Some non-metric features were scored using the Arizona State University Dental Anthropology System (ASUDAS) [56].

Mesiodistal (MD) and buccolingual (BL) dimensions of the crown, as well as root length (from the cemento-enamel junction [CEJ] to root tip) were taken with a standard sliding caliper and recorded to the nearest 0.1 mm following the methods of Wolpoff [57]. Bi-variate plots of the MD and BL diameters will be provided for the metric comparison of Hexian with other hominin samples.

### 

#### Microcomputed tomography, reconstructions of the enamel dentine junction (EDJ) surface and pulp cavity, and measurement of tissue proportions

The heavy occlusal wear of most of the Hexian teeth has erased many relevant features of the outer enamel surface (OES). In order to complement the external morphological descriptions, digital three-dimensional surface models of the EDJ were reconstructed from the high resolution microcomputed tomography (micro-CT) scanning. Among Hexian teeth, one M_2_ (PA834-1) is severely worn, and the I^1^ (PA835) and one of the M_2_s (PA838) were not available for micro-CT scanning. Therefore, the EDJ surfaces of all Hexian teeth but these three specimens will be provided. In addition, the pulp cavity was also reconstructed for morphological description and volumetric measurement. Although the comparative material for the EDJ and pulp cavity is very limited, we provide these measurements to better characterize the Hexian population and to allow future comparisons. The reconstruction of the pulp cavity was only performed on teeth with complete and well-preserved root (i.e., PA832 and PA834-2). Each tooth was scanned using a 225 kV-µCT scanner (designed by Institute of High Energy Physics, Chinese Academy of Sciences, and housed at the Institute of Vertebrate Paleontology and Paleoanthropology, Chinese Academy of Sciences) equipped with a 1.0-mm aluminum-copper filter, under settings of 120–150 kV, 120 uA, 0.5 rotation step, 360 degrees of rotation and 4 frame averaging. Isometric voxel size is 12.5–18.8 microns. The settings varied with the size of the objects and their fossilization degree. Raw projections were converted into image stacks of raw file format (tomographic slices) with IVPP225kVCT_Recon. The image stacks are stored at a resolution of 2048×2048 pixels. VG studio Program was employed to remove the empty spaces from the image stack in order to reduce the data size and to save the data as the format of raw volume. They were then imported into Mimics 16.0 to complete the segmentation of the enamel and the dentine and to visualize the EDJ surfaces and pulp cavities. During the processes of enamel/dentine segmentation and reconstruction of the pulp cavity, 3D Livewire, single/multiple slices modification, and thresholding were employed.

**Figure 3:**
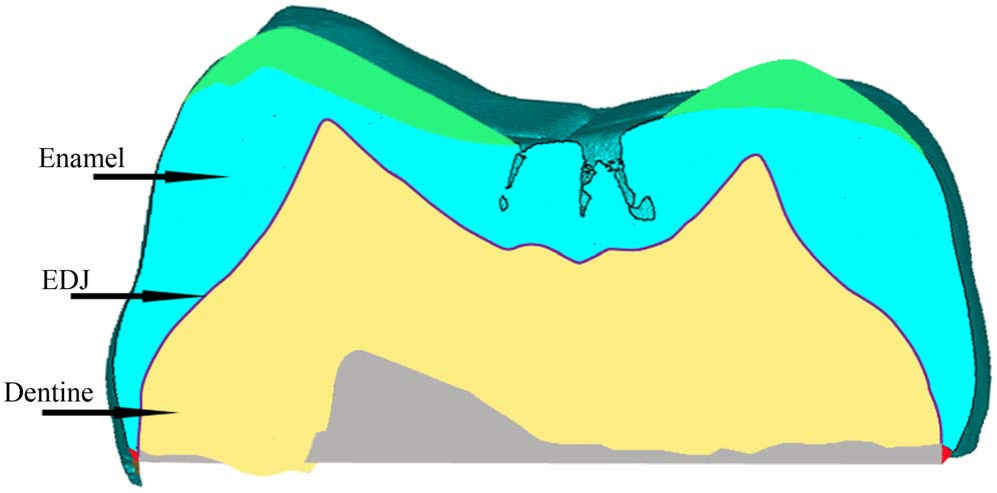
The mesial sectional plane of Hexian PA833 (the red and grey areas indicate the reconstructed enamel and dentine, respectively, and green areas show how the occlusal wear was virtually restored).

Besides the reconstructions of the EDJ surface and the pulp cavity, we also calculated the tissue proportions of one of the M^2^s (PA833). Unfortunately, the crowns of Hexian P^3^, M^1^, M_2_, and M_3_ are too worn to perform this analysis and one of the M^2^s (PA837) is partially broken along the cervical line, especially in its buccal aspect. PA833 also lacks a minimal part of its enamel and dentine along the cervical line, but we reconstructed it with Mimics 16.0 based on the orientation of the outer enamel and inner EDJ surfaces (Fig. 3). Besides, the occlusal wear was also corrected by virtually reconstructing the missing enamel portion employing unworn occlusal surfaces as models. The average and relative enamel thickness of PA833 were measured on a 2D sectional plane of the crown. The corrected and uncorrected values will be provided. The plane was prepared following Olejniczak [58]. Firstly, a horizontal plane was defined by three dentine horn tips (paracone, metacone, and protocone). Then, we defined a sectional plane, perpendicular to the previous one and that was simultaneously cutting through the dentine horn tips of paracone and protocone. The enamel cap (c) and dentine (b) areas and the length of EDJ line (e) were measured. The dentine area here was defined as the one enclosed by the EDJ line and the shortest connection line between the two cervical points. The section of the mesial plane and the measurements were performed with 3-matic. Average enamel thickness (AET) was defined as c/e, and relative enamel thickness (RET) as 100×AET/√b. Hexian variables were compared with those obtained by Olejniczak et al. [59] and Smith et al. [60]. Smith et al. [60] employed an alternative method to define the mesial sectional plane. First, they pinpointed the 3D midpoint between the pulp horn tips of the paracone and the protocone and then, the sectional plane was defined through the dentine horn tips. No significant differences were found between the AET and RET obtained with both methods [60]. Therefore, the data acquired with both protocols can be used in an inter-species comparison.

## Description of the Hexian Teeth

### Right upper third premolar (P3 PA832) (Fig. 4)

In general the tooth is in good condition, except for several enamel cracks that extend along all the crown surfaces. There is some alveolar bone still attached to the root. Previous studies have reported this tooth as an upper fourth premolar [4]. However, in the present study, we have reclassified it as an upper third premolar based on the identification of some morphological features that are useful to discriminate a P^3^ from a P^4^, such as a deep depression or mesial concavity in the mesial aspect of the crown and the root, and the fact that the round-shaped mesial interproximal facet is smaller than the distal one and fits with the contact pattern with a canine. Besides, the morphology of the socket fragment attached to the mesial aspect of the root recalls the canine root shape. Finally, and as it will be explained below, the expression of two buccal radicals is more frequent in P^3^s than in P^4^s.

The occlusal wear has flattened both buccal and lingual cusps. According to Molnar [51], the band shaped dentine corresponds to a wear stage 4.

From the occlusal view, the crown outline is oval and slightly asymmetrical. The buccal and lingual cusps are partially connected by a thin transverse crest that obliterates the mesial aspect of the central groove. Mesiobuccal and distobuccal grooves originate from the anterior and the posterior foveae. Because of the occlusal wear, the ridge pattern of the buccal cusp is unclear. Viewed from the buccal aspect, the crown is relatively symmetrical. The enamel surface is relatively smooth and featureless.

**Figure 4:**
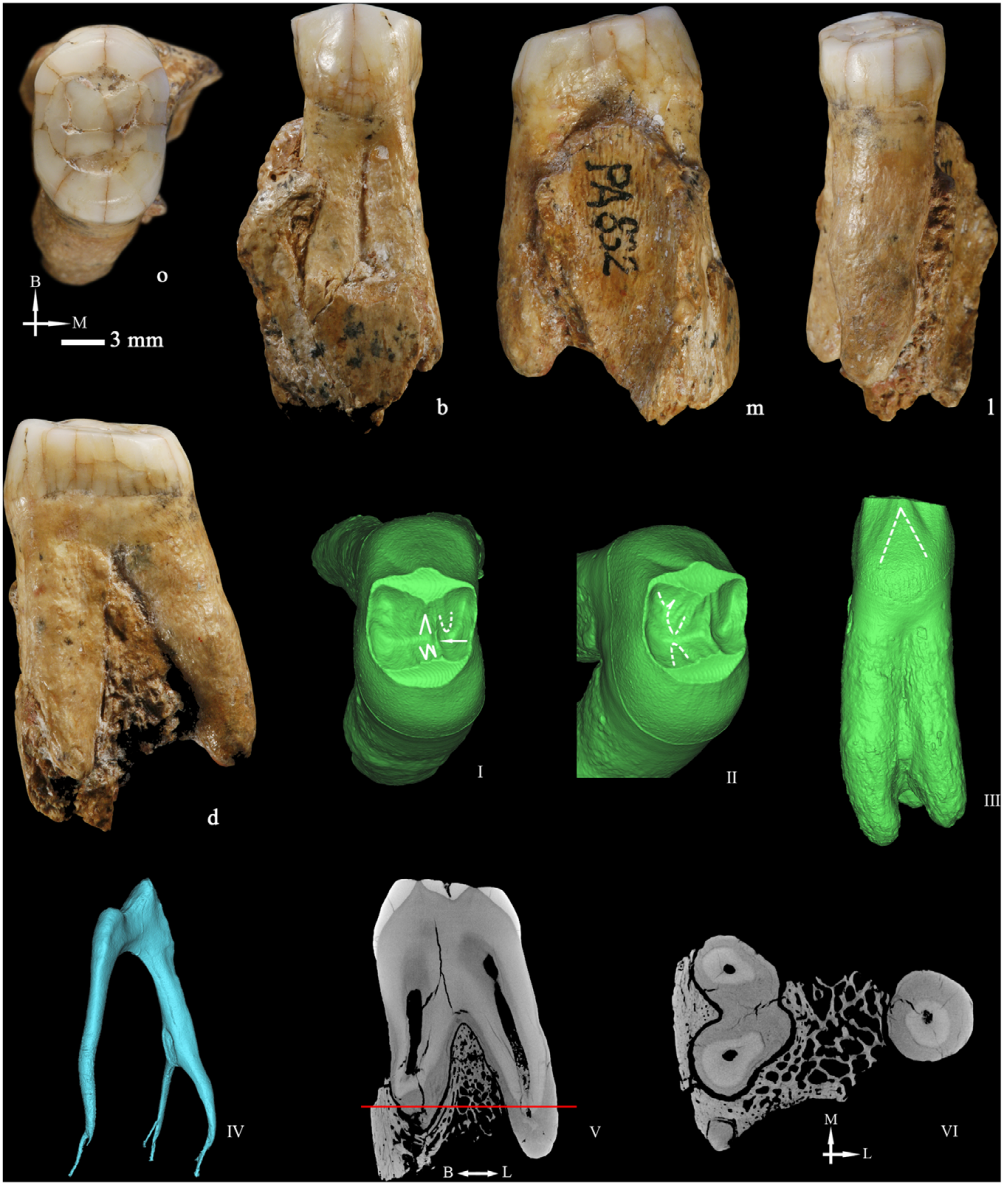
Right upper third premolar (PA832) (o: occlusal, B or b: buccal, M or m: mesial, L or l: lingual, d: distal). 3D reconstructions of the dentine surface (I–III) and the pulp cavity (IV) obtained from micro-CT scanning. The arrow points to the transverse crest. Solid lines indicate the bifurcation of the essential crest. Dotted lines indicate the mesial (I) and distal accessory ridges (II) and the well-demarcated central ridge on the buccal surface (III). Sagittal sectional plane of PA832 (V). Cross-section of PA832 (VI) at the level indicated by the red line. I, II, III, IV, V, and VI are not scaled.

At the EDJ (Fig. 4I and II), the essential crest of the buccal cusp bifurcates into two branches, and the mesial one forms the buccal component of the transverse crest. There is a mesial accessory ridge and three slightly elevated distal accessory ridges. The lingual essential crest [61] is composed by three ridges and distal to it, there is a slightly elevated distal accessory ridge. The marginal ridges are thick but no accessory tubercles are expressed. In the buccal surface, a faint longitudinal mesial depression and a moderately deep distal furrow can be identified (Fig. 4III). Moreover, the basal region of the crown buccal surface bulges slightly forming a cingulum that it is not obvious at the OES.

PA832 presents three roots, two buccal and one lingual that separate from the apical quarter. The lengths of the mesiobuccal, distobuccal, and lingual roots are 19.26, 17.88, and 18.96 mm, respectively. The virtual removal of the alveolar bone attached to them, reveals that the buccal roots are separated from the second third to the tip, although they are joined by a lamina of cementum/dentine until the apical third (Fig. 4III). The roots are remarkably robust, with a round section that barely narrows towards the tip. The tomographic slices acquired from micro-CT scanning show that the cementum is uniformly overlaid on the dentine surface so the thickness is not due to a pathological deposition of the cementum (Fig. 4V and VI). The roots are widely divergent and their projection can be seen from the occlusal aspect.

The pulp horns of PA832 (Fig. 4 IV) are relatively blunt and low. There is a buccal and a lingual canal, with the former bifurcating into a mesial and distal branch from the apical half. At the apical end, small secondary canals develop. The total volume of the pulp cavity is 82.67 mm^3^.

### Left upper third premolar (P3 HXUP3) (Fig. 5)

The crown is complete but the root is broken, and it only preserves a small distal fragment of about 6 mm to the cervical line. The occlusal wear has flattened the cusp apices exposing two large patches of dentine that correspond to a grade 4 [51]. There are two oval interproximal wear facets on both the mesial and the distal aspects of the crown, with the distal one being larger than the mesial one.

From the occlusal view the crown outline is a slightly asymmetrical oval. The buccal and lingual cusps are completely separated by the central groove. The heavy occlusal wear has erased most of the OES features. On the buccal aspect of the crown, a pronounced mesial vertical furrow can be distinguished, while the distal furrow is weakly developed and runs towards the apex of the cusp.

**Figure 5:**
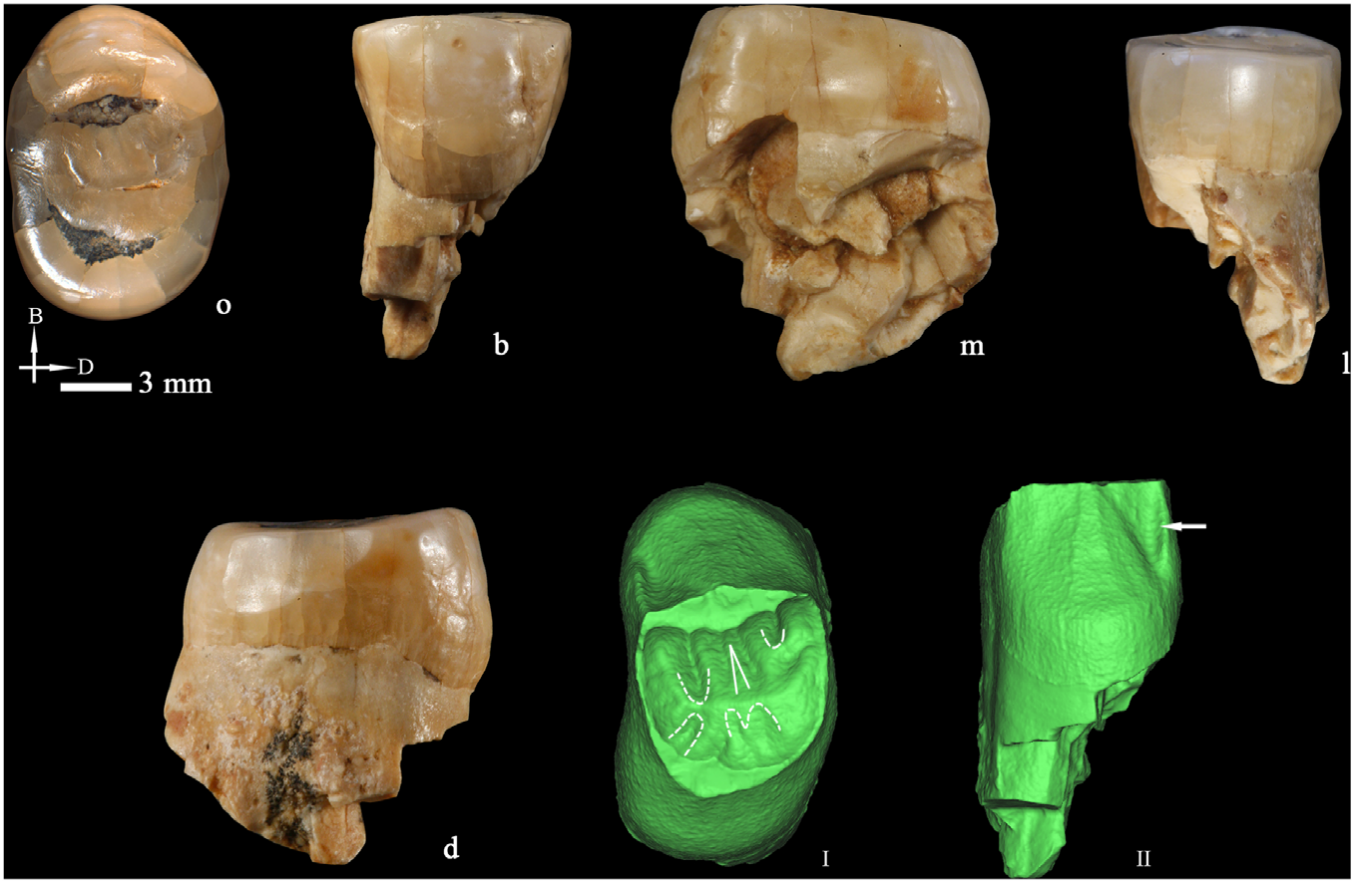
Left upper third premolar (HXUP3) (o: occlusal, B or b: buccal, m: mesial, l: lingual, D or d: distal). The occlusal (I) and buccal (II) views of the dentine surface reconstructed from micro-CT scanning. Solid lines indicate the bifurcation of the essential crest. Dotted lines indicate mesial and distal accessory ridges. The arrow points to the vertical groove on the buccal surface. I and II are not scaled.

At the dentine, the buccal essential crest is bifurcated and we distinguish a mesial and a distal accessory ridge. The lingual essential crest is single, and also a mesial and a distal accessory ridge are expressed (Fig. 5I). On the mesial aspect of the buccal surface, there is a deep furrow that demarcates a strong mesial ridge (Fig. 5II). The cervical region of the buccal surface bulges and forms a slight cingulum.

The root is broken so the exact number of radicals and their morphology cannot be determined. However, from the fragment of root preserved we can see the beginning of a bifurcation in the distal surface meaning that this tooth had at least a buccal and a lingual root.

### Left upper first molar (M1 PA836) (Fig. 6)

The preservation is good except for the buccal roots, which are broken from the cervical line. The occlusal surface is worn, corresponding to a grade 5 [51]. The interproximal wear facets are oval-shaped and large.

From the occlusal aspect, the shape of the crown outline is approximately square with a moderately oblique buccal contour. Wear has obliterated most of the enamel morphological features, but the four main cusps can still be discerned by the remnant of the occlusal grooves and the pattern of the dentine exposed. The hypocone is large (ASUDAS grade 4).

**Figure 6:**
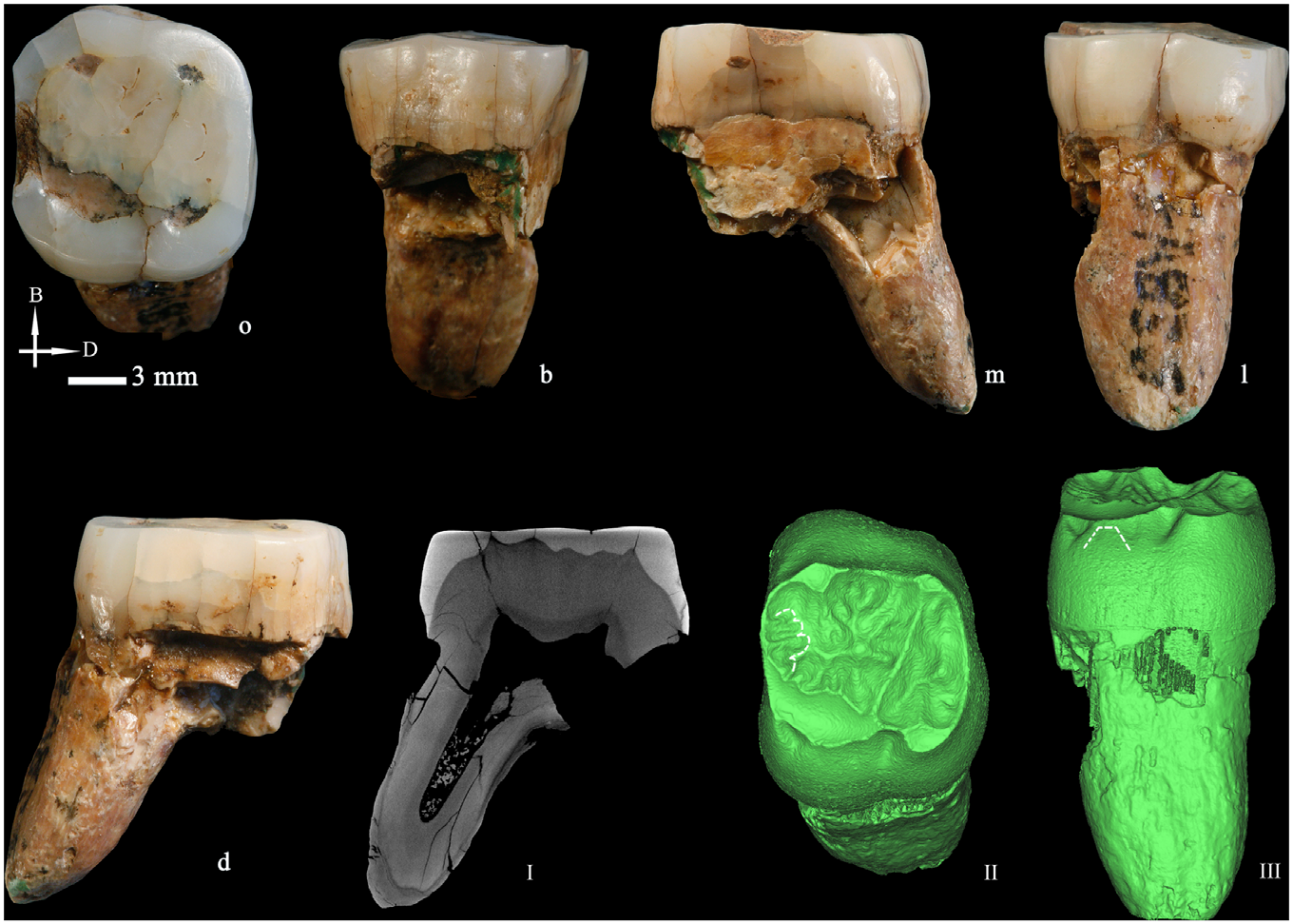
Left upper first molar (PA836) (o: occlusal, B or b: buccal, m: mesial, l: lingual, D or d: distal). Sagittal sectional plane of PA836 (I). The occlusal (II) and lingual (III) views of dentine surface reconstructed from micro-CT scanning. Dotted lines point to the expression of mesial accessory ridges (II) and a Carabelli’s trait (III). I, II, and III are not scaled.

The dentine surface is strongly crenulated and complex and displays several crests and ridges on all cusps (Fig. 6II). There are five ridges at the mesial marginal ridge. The protocone and the metacone are connected by a high and continuous *crista obliqua*. Mesial to the lingual groove, a weak and a deep furrow define a Carabelli’s cusp, which is hard to score because it is affected by wear (Fig. 6III).

The preserved lingual root is very robust and bucco-lingually flattened with a rounded tip. It strongly diverges lingually. Viewed on the tomographic slices, there is no abnormal cementum deposition deforming the root (Fig. 6I). The lingual root is 14.72 mm long from the cervical line to the tip.

### Left upper second molar (M2 PA833) (Fig. 7)

The entire root is missing, and the crown is slightly damaged along the cervical line. The occlusal wear has flattened the lingual cusps without exposing the dentine, corresponding to Molnar’s stage 2 [51]. On both the mesial and the distal sides of the crown there is an oval-shaped interproximal facet. The mesial one is small and the distal one is medium-sized.

**Figure 7:**
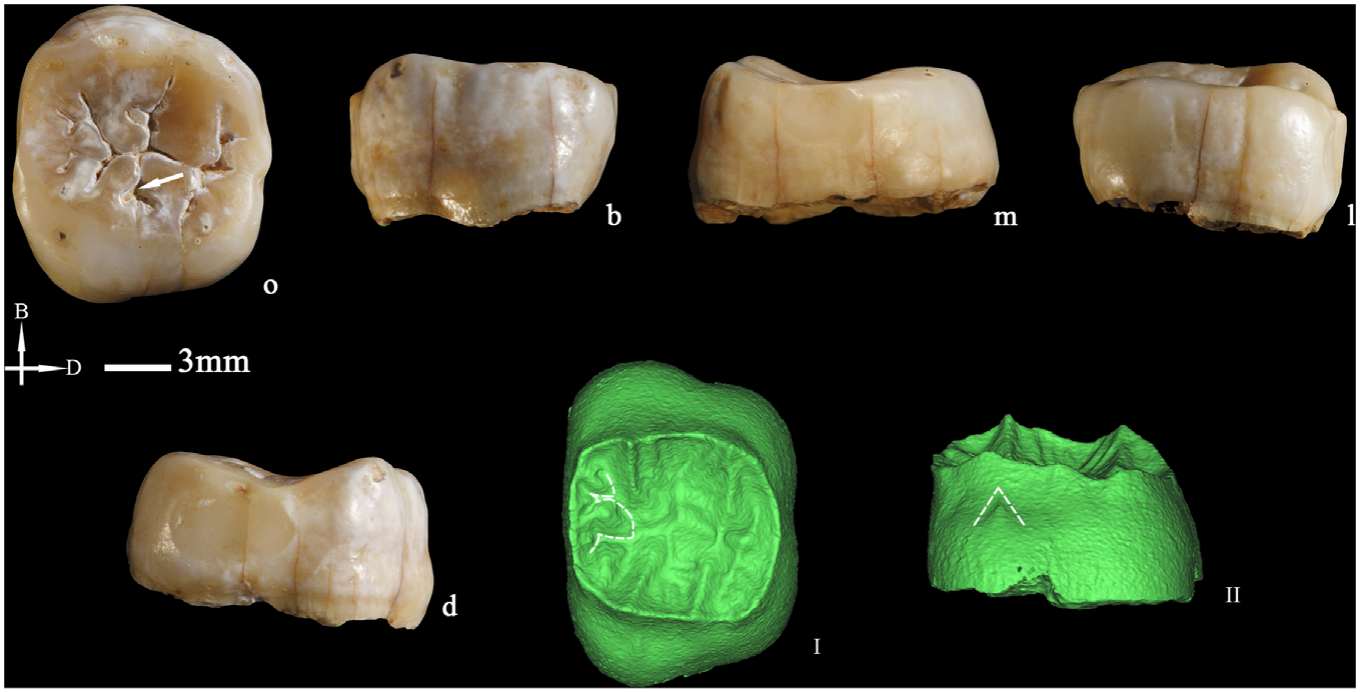
Left upper second molar (PA833) (o: occlusal, B or b: buccal, m: mesial, l: lingual, D or d: distal). The occlusal (I) and lingual (II) views of dentine surface reconstructed from micro-CT scanning. Dotted lines point to the mesial accessory ridges (I) of the occlusal surface and to the expression of a Carabelli’s cusp on the lingual face of the crown (II). I and II are not scaled.

The occlusal outline is trapezoidal with a narrower distal half. On the occlusal surface, the four main cusps can be distinguished. The hypocone is medium-sized (ASUDAS grade 3.5) and there is a C5 of ASUDAS grade 3. The surface of the four main cusps is crossed by several secondary grooves. The lingual prolongation of the buccal groove is particularly pronounced, and divides the protocone into two parts (Fig. 7o). Besides, there are four mesial accessory ridges. No transverse crest [55] and *crista obliqua* are expressed. The mesiolingual surface of the crown is relatively smooth.

The dentine surface is complicated by the bifurcation of the essential crests and the expression of several accessory ridges (Fig. 7I). The *crista obliqua* are discontinuous. There are four mesial accessory ridges, with the largest one almost having a free apex. The distal marginal ridge is continuous and smooth, without signs of a C5 despite its identification at the OES. At the mesiolingual aspect of the crown, there is a weak V-shaped elevation that corresponds to a grade 5 of Carabelli’s trait (Fig. 7II) [56], [62]. Although the buccal and lingual sides are relatively vertical at the OES, at the EDJ they bulge strongly at the level of the protocone and the paracone.

The enamel cap area on the mesial section of PA833 was 25.71 mm^2^ and 29.81 mm^2^ before and after correcting for occlusal wear, respectively. Dentine area was 41.22 mm^2^, and the length of EDJ line was 19.75 mm. Therefore, the corrected average enamel thickness (AET) and relative enamel thickness (RET) of PA833 are 1.51 mm and 23.52 mm, respectively. The uncorrected values are 1.30 mm for the AET and 20.28 for the RET.

### Right upper second molar (M^2^ PA837) (Fig. 8)

Like PA833, this tooth only preserves the crown and its root is broken from the cervical region. The crown is partially broken along the cervical line, especially in the buccal and mesial aspects. The occlusal wear has flattened the lingual cusps without exposing the dentine and corresponds to stage 2 [51]. There are two oval interproximal wear facets, the mesial one being larger than the distal one.

**Figure 8:**
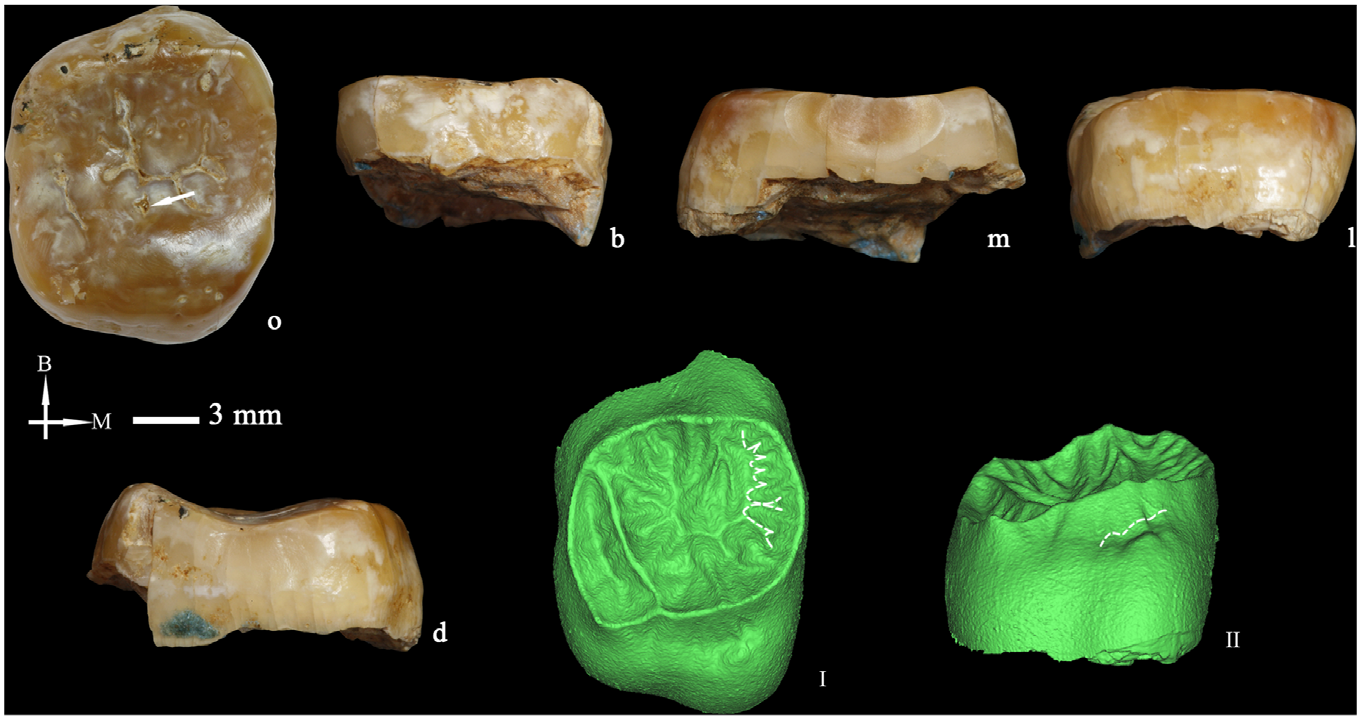
Right upper second molar (PA837) (o: occlusal, B or b: buccal, M or m: mesial, l: lingual, d: distal. I and II: The occlusal (I) and lingual (II) views of dentine surface reconstructed from micro-CT scanning). Dotted lines point to the mesial accessory ridges of the occlusal surface and to the expression of a Carabelli’s cusp on the lingual surface of the crown. I and II are not scaled.

The shape of the crown outline resembles that of PA833. It is trapezoidal with the distal half narrower than the mesial half. The hypocone is medium-sized (ASUDAS grade 3.5). No cusp 5 is detected. A transverse crest is absent. There are several secondary grooves. The prolongation of the buccal groove is particularly pronounced and divides the large protocone into two portions (Fig. 8o). The metacone essential crest connects with the distal aspect of the protocone and forms a high and continuous *crista obliqua*. At the mesial marginal ridge, there are at least four accessory ridges. On the buccal surface of the paracone, there are faint vertical grooves that delimit a small elevation. On the lingual and mesial sides of the protocone, there are several pit-like features.

At the EDJ (Fig. 8I), there are no traces of a C5 or a transverse crest. The occlusal surface is highly crenulated due to the expression of several secondary grooves and ridges. At the mesial aspect, there are up to eight accessory ridges although none of them reach a tubercle-like aspect. Carabelli’s trait is represented by three elevations, with the middle one having a free apex (Fig. 8II) (ASUDAS grade 7) [56], [62]. The buccal and lingual surfaces of the protocone and the paracone are remarkably projected laterally.

### Left lower second molar (M_2_ PA834-1) (Fig. 9)

There are two left lower molars (PA834-1 and PA834-2) that belong to the same individual and are attached through a small fragment of alveolar bone. In an initial report [4] the teeth were reported as lower first and second molars. In the present study, we have reclassified them as second and third molars, particularly because of the elongated and distally narrowed aspect of the M_3_ and the lack of a distal interproximal facet.

There is some enamel damage on the mesiolingual corner of the crown of PA834-1 and the mesial roots also present some crack lines. The tooth is severely worn and corresponds to stage 6 [51]. There is a small interproximal wear facet on the mesial side.

**Figure 9:**
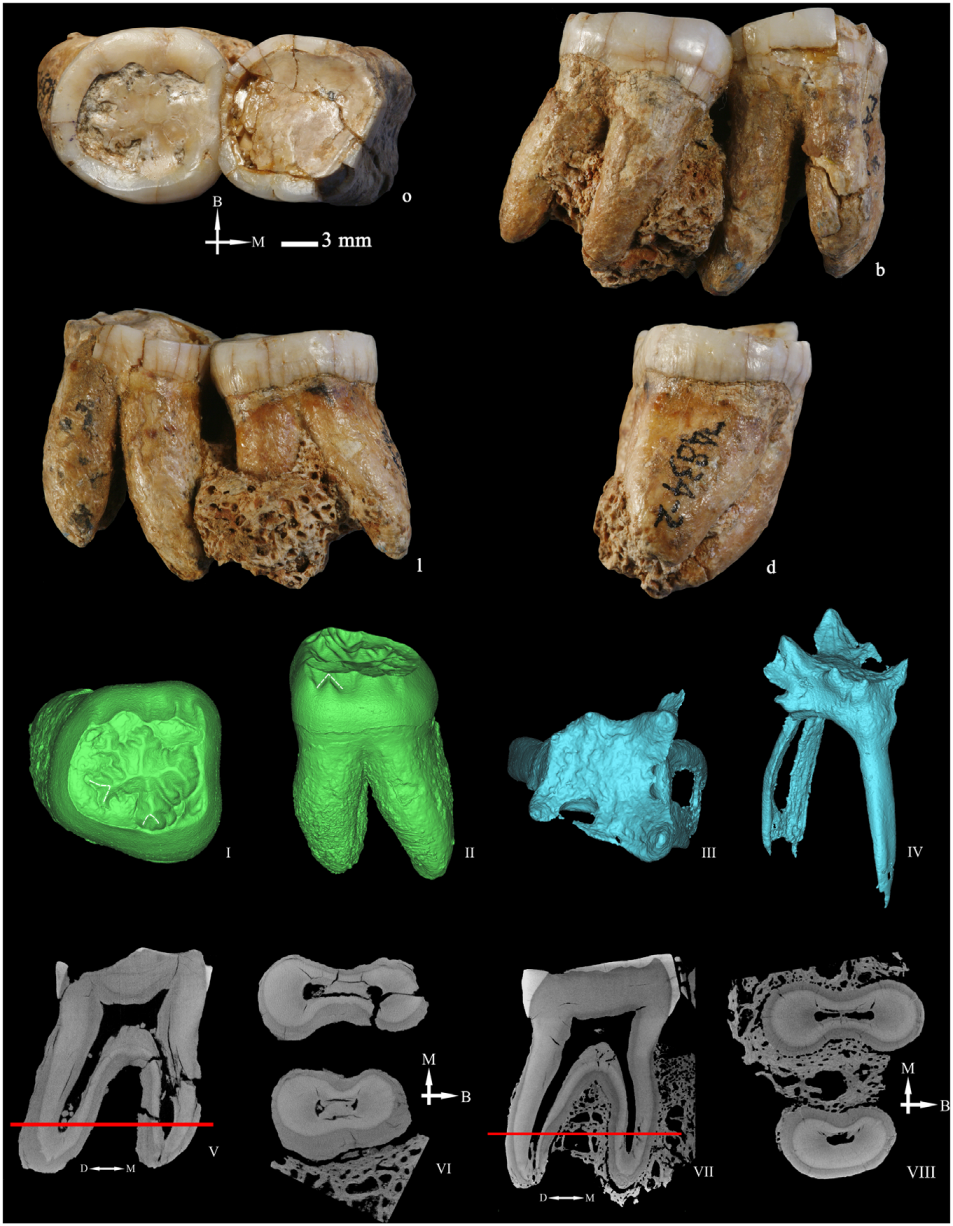
Left lower second and third molars (PA834-1 and PA834-2) (o: occlusal, B or b: buccal, M: mesial, l: lingual, d: distal). The occlusal (I) and lingual (II) views of dentine surface reconstructed from micro-CT scanning of PA834-2. The occlusal (III) and distobuccal (IV) views of the pulp cavity of PA834-2 reconstructed from micro-CT scanning. Sagittal sectional planes of PA834-1 (V) and PA834-2 (VII). Cross-section of PA834-1 (VI) and PA834-2 (VIII) at the level indicated by the red lines. Dotted lines indicate the Cusp 6 and 7 on the occlusal surfaces and the protostylid on the buccal surfaces of the crown. I, II, III, IV, V, VI, VII, and VIII are not scaled.

The crown outline of PA834-1 is approximately rounded and its BL dimension is slightly larger than the MD dimension. Because of the severe wear, we cannot assess any relevant occlusal morphological feature. The tooth has mesial and distal roots that are mesiodistally compressed. Each root has a buccal and a lingual component demarcated by a shallow longitudinal depression. The roots look robust and bifurcate close to the cement-enamel junction, although the micro-CT image reveals that the separation of the canals is higher (Fig. 9V). The mesial and distal roots are 15.07 and 16.84 mm long, respectively. The breadths of both roots are close to the BL dimension of the crown and they only narrow at the level of the root tips. The micro-CT slices show that the cementum deposition is smooth (Fig. 9V and VI). Due to the mesial fracture, the 3D structure of the entire root cannot be reconstructed but the mesial root has two independent canals that run all along the entire root length till the tip (Fig. 9VI). The distal root canal is single with a bifid tip.

### Left lower third molar (M_3_ PA834-2) (Fig. 9)

The tooth is well preserved with only small enamel damage on the occlusal surface. Occlusal wear has flattened the cusps and exposed several coalescing patches of dentine that correspond to a stage 5 [51].

The occlusal contour is oval-shaped with a slightly narrower distolingual aspect. Occlusal wear has erased most of the features at the OES. The hypoconulid is buccally displaced. Two small grooves start from the distal fovea and define a medium-sized C6. Grooves are arranged in a “X” pattern. There are minor enamel extensions along the cement-enamel junction (CEJ) in the buccal and lingual sides (ASUDAS grade 1). At the OES there are no signs of protostylid.

The dentine surface (Fig. 9I) is remarkably complicated by the expression of bifurcated essential crests and multiple accessory ridges. There is an elevated ridge at the location of the C6. Besides, there are two small ridges between the metaconid and the entoconid that slightly protrude in the lingual contour and could correspond with a C7. There are five small accessory ridges at the mesial marginal ridge and no signs of middle trigonid crest. At the EDJ, the protostylid is expressed as prominent triangular elevation without free apex (Fig. 9II) (ASUDAS grade 6). According to the standards proposed by Skinner et al. [63] at the dentine surface, the protostylid of PA834-2 is restricted to the “middle region” of the buccal face. Mesial to the protostylid, there is another dentine shelf that corresponds to a prolongation of the mesial marginal ridge onto the buccal surface. Hlukso [64] described this structure as mesial protoconid ridge because this structure did not covary with the protostylid expression. However, Skinner et al. [63] were more inclined to include this feature within the protostylid because their studies suggested that the same developmental processes were underlying these two features.

Like the M_2_ (PA834-1), this tooth also has two roots that bifurcate at the cervical third and diverge towards distal. However, the root canals bifurcate at a higher level (Fig. 9 VII). The mesial and distal roots are 14.52 and 15.56 mm long, respectively. The roots are robust and mesio-distally compressed. They maintain their width along their length, only narrowing at the tip. The micro-CT sagittal and cross-sectional planes show that the root tips are evenly capped by the cementum (Fig. 9VII and VIII).

The coronal pulp of PA834-2 (Fig. 9III and IV) has five horns that correspond with each of the five main cusps. There are three root canals, two mesial and one lingual. The mesial canals coalesce in the apical fourth but with bifid apices. The volume of the pulp cavity is 70.54 mm^3^.

### Left lower second molar (M_2_ PA839) (Fig. 10)

The entire root is missing and except for some enamel damage on the distal surface, the crown is well preserved. The cusps are flattened by the occlusal wear and large patches of dentine are exposed on the mesial portion corresponding to a grade 4 [51]. Interproximal wear facets can be observed on both mesial and distal sides of the crown. The mesial one is represented by two small oval-shaped depressions, and the distal one is affected by the enamel fracture.

The crown outline shape of PA839 is an asymmetrical oval with a tapering distobuccal half in contrast with a strongly expanded mesiobuccal contour. In addition to the five main cusps, PA839 expresses a C6 and a C7. The metaconid and the hypoconid are in contact defining a Y-shaped groove pattern. The hypoconulid is large (ASUDAS grade 4) and buccally displaced. Part of the C6 is damaged, but from the orientation of the preserved grooves we can assess that is medium-sized and smaller than the hypoconulid (ASUDAS grade 2). The C7 is also medium-sized (ASUDAS grade 4). There are several secondary grooves dividing the essential ridges of the main cusps. The essential crest of the metaconid is deflected in a right-angle but does not reach the entoconid so it corresponds to a deflecting wrinkle of ASUDAS grade 2. The severe wear obscures the morphology of the mesial marginal ridge but there are no sign of middle trigonid crest. On the buccal surface of the protoconid, and mesial to the mesiobuccal groove, we observe a deep pit and several enamel depressions, which would correspond to a protostylid (at least ASUDAS grade 5).

**Figure 10:**
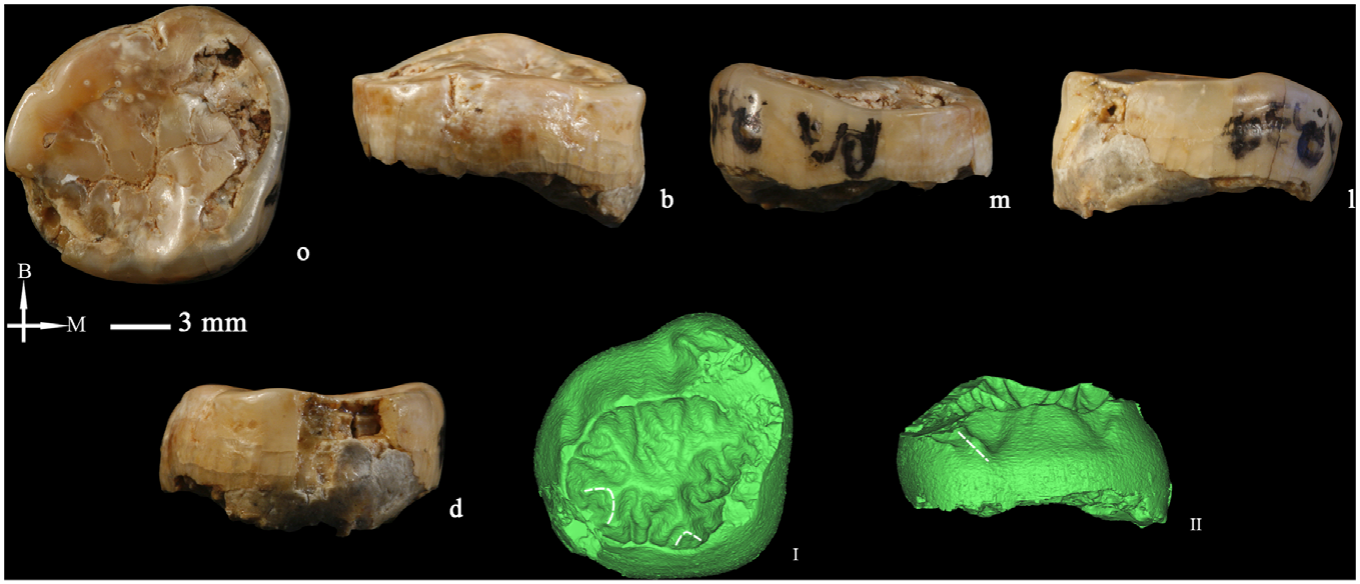
Left lower second molar (PA839) (o: occlusal, B or b: buccal, M or m: mesial, l: lingual, d: distal. I and II: The occlusal (I) and buccal (II) views of the dentine surface reconstructed from micro-CT scanning. Dotted lines indicate the Cusp 6 and 7 on the occlusal surfaces and the protostylid on the buccal surfaces of the crown. I and II are not scaled.

The main and accessory cusps identified at the OES can also be discerned at the EDJ (Fig. 10I). The occlusal surface is highly crenulated and complex, with several ridges and bifurcated essential crests. The occlusal projection of the buccal and the lingual surfaces at the level of the protoconid and the metaconid is notable. The protostylid at the EDJ (Fig. 10II) is identified as a pronounced cleft without free apex and classified as a grade 5 or 6 according to ASUDAS. Mesial to the protostylid, there is a dentine shelf that corresponds to the projection of the mesial marginal ridge onto the buccal surface.

### Left lower second molar (M_2_ PA838) (Fig. 11)

The tooth is well preserved, with only superficial damage at the apical third of the buccal surface of the mesial root. The occlusal wear has flattened all cusps, leaving exposed large patches of dentine that correspond to grade 4 [51]. On both the mesial and the distal sides of the crown, large oval-shaped interproximal wear facets can be identified.

PA838 has an oval crown outline, where the distal half is slightly narrower than the mesial half. It expresses the five main cusps and the hypoconulid occupies a buccal position. The occlusal wear complicates the identification of both the C6 and the deflecting wrinkle. The main cusps are arranged in a “Y” pattern. The central groove bifurcates at the anterior fovea and there are no signs of a middle trigonid crest. The metaconid crest is deflects distally and contacts the entoconid, corresponding to a deflecting wrinkle of ASUDAS grade 3. On the buccal side, there is a slight enamel extension that corresponds to ASUDAS grade 1.

The root is remarkably robust and bifurcates into a mesial and a distal component. The bifurcation is higher in the lingual side (upper third) than in the buccal side (middle third). The mesial and distal roots are 15.96 mm and 16.00 mm long, respectively. Both roots are mesio-distally compressed but only taper at the apices when viewed laterally.

### Right upper central incisor (I^1^ PA835) (Fig. 12)

Except for a small fracture at the root tip, this tooth is in excellent condition. Occlusal wear only affects the incisal edge and corresponds to grade 2 of Molnar [51]. Oval-shaped and medium-sized interproximal wear facets can be observed on both the mesial and the distal sides of the crown.

From the occlusal aspect, the labial surface is moderately convex at its basal part (ASUDAS grade 3) while relatively flat towards the incisal edge.

From the lingual aspect, the mesial and distal marginal ridges are thickened corresponding to a shovel shape of ASUDAS grade 4. There is a round and bulging basal eminence. Circumscribed to it, several secondary grooves delimit the expression of five small finger projections (tuberculum dentale of ASUDAS grade 4). The labial surface is not smooth and it is crossed by three deep vertical grooves that delimit a wide central and two lateral ridges that correspond with the labial reflection of the thickened marginal ridges.

The root is robust and short and it is slightly mesio-distally compressed. Both the buccal and the lingual sides are convex. Shallow longitudinal grooves run along its mesial and distal surfaces, with the distal one being deeper.

## Comparative Morphology (See Also S4 Table in S1 Text)

### Upper third premolar (P^3^)

The crown features of the Hexian P^3^s are not particularly useful from a taxonomic point of view. These specimens are variable in the expression of the transverse crest and vertical grooves at the buccal surface. While in HUXP3 the crest is absent, in PA832 it is thin but continuous; and while HUXP3 develops a prominent vertical groove at the mesial aspect of the buccal surface, these buccal grooves are absent in PA832. The occurrence of a transverse crest in the P^3^s is relatively high in *Australopithecus* and African early *Homo* groups [35], [36], [65], decreasing its frequency in late *Homo* specimens. However, the frequencies are slightly higher in East Asian Early Pleistocene and European Middle Pleistocene hominins [43], [66]. Though this feature can also be found in specimens from other taxonomic groups, the cases are relatively rare [43], [52], [55].

On the buccal surface, the mesial vertical groove is generally deeper than its distal counterpart. There is a progressive tendency to express shallower and weaker mesial and distal grooves throughout the genus *Homo* [18] with some fossil hominins from the European Middle Pleistocene, Neanderthals, and early and recent modern humans showing smooth buccal surfaces [18], [43]. The prominent vertical groove on the buccal face of HXUP3 would be more typical of East Asian Early and some mid-Middle Pleistocene hominins such as those from Zhoukoudian Locality 1 [18]. In contrast, this type of groove in *H. ergaster* ranges from weak to moderate.

The asymmetrical oval crown outlines of the Hexian P^3^s resemble those of *Australopithecus*, African Early *Homo*, *H. ergaster*, and hominins from Sangiran Dome, Zhoukoudian, and Chaoxian [17], [19], [67]. Interestingly, one of the Asian Late Middle Pleistocene specimens (Panxian Dadong) is morphologically closer to *H. sapiens* by exhibiting a relatively symmetrical crown outline with a much wider buccal aspect [19].

In contrast to the crown, the morphology of the root is in this case more informative. Among the fossil hominins, three-rooted P^3^s like PA832 are unusual and only found in early taxonomic groups like *Australopithecus*, African early *Homo*, and fossil hominins from East Asian Early Pleistocene (e.g., S-4, S7–35, and S7–36, see also Fig. 13) [16], [65], [66], [68]–[70]. Indeed, the P^3^s from Zhoukoudian Locality 1 have only two roots [52] (Fig. 13). In addition, the roots of Hexian P^3^ do not show the gradual narrowing we usually find in African and European *Homo* specimens. Instead, it maintains a round and wide section along the root length until the tip, which is typical of premolars of East Asian Early Pleistocene fossils such from Sangiran (Pucangan) or Jianshi [71].

**Figure 11:**
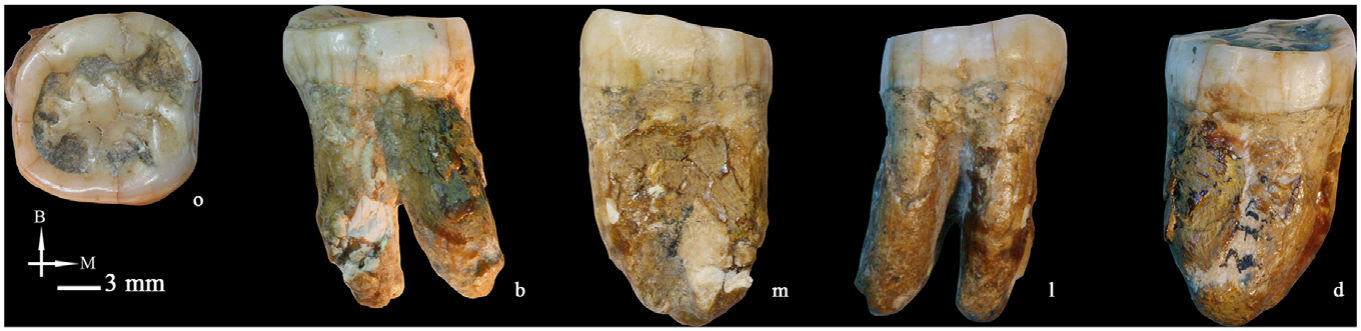
Left lower second molar (PA838) (o: occlusal, B or b: buccal, M or m: mesial, l: lingual, d: distal).

**Figure 12:**
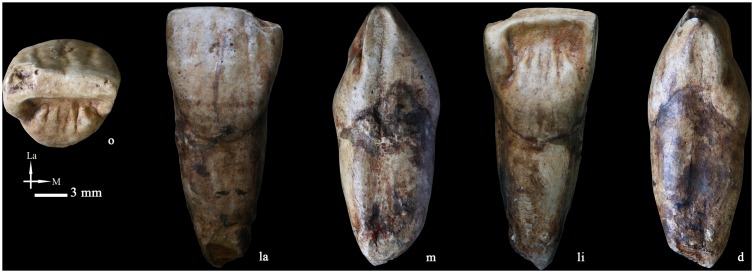
High quality replica of the right upper central incisor (PA835) (o: occlusal, La or la: labial, M or m: mesial, li: lingual, d: distal).

**Figure 13:**
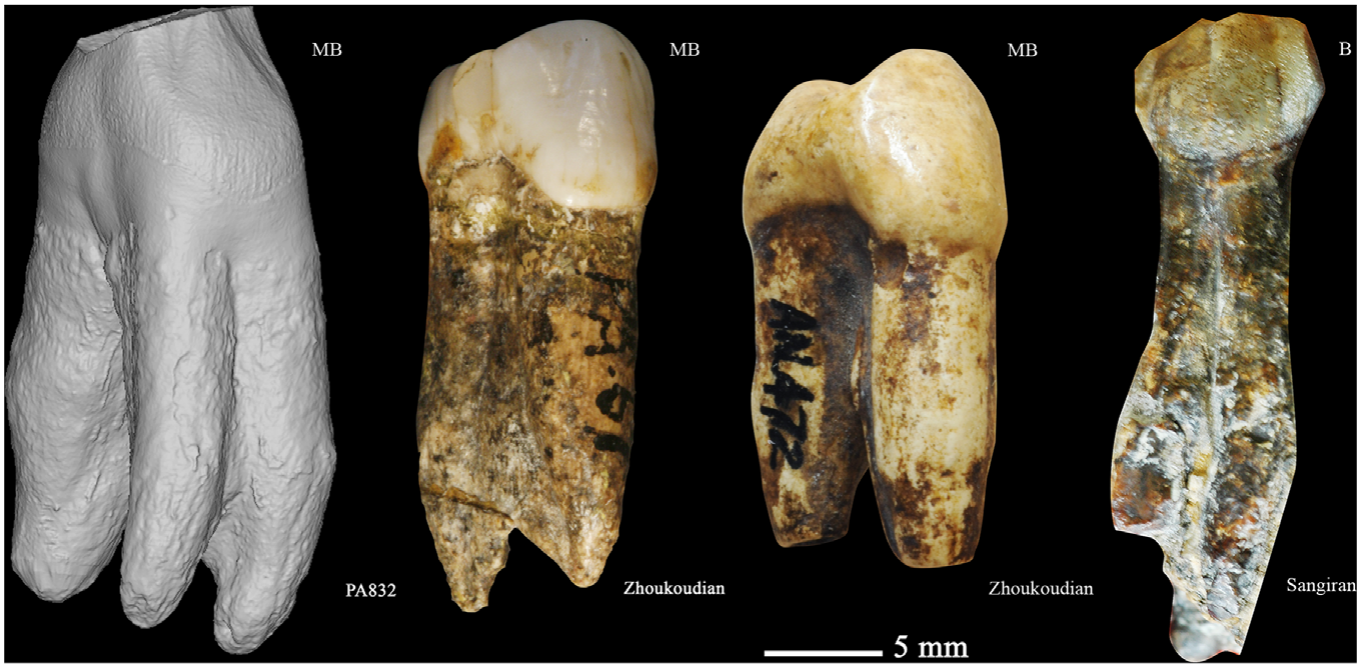
From left to right, 3D reconstruction of Hexian PA832 from micro-CT scanning, Zhoukoudian PA67, Zhoukoudian 19, and Sangiran S7–35 (MB: mesiobuccal, B: buccal).

### Upper first molar (M^1^)

Though the present study did not perform a metric analysis of the cusp size, the ASUDAS [56] plaque for the hypocone size can be employed to assess some trends within groups. The hypocone size tends to decrease throughout hominin evolution [41], [72]. Generally speaking, *Australopithecus* and Early and Middle Pleistocene *Homo* tend to have large or very large hypocones ([14], [18], [72], [73]; but see [74]). The large hypocone exhibited by the Hexian M^1^ fits within the typical morphologies of the Middle Pleistocene fossils in general and in particular of those from East Asia. However, the Hexian M^1^ could be differentiated from those of Zhoukoudian Locality 1, since the latter tends to express medium-sized hypocones [18]. The crown outline of Hexian M^1^ is trapezoidal with an oblique buccal contour. It is a shape typical in most hominins from Sangiran, Zhoukoudian, and Chaoxian, and differentiated from early Pleistocene fossils from Africa, European Early and Middle Pleistocene hominins, and Neanderthals, which show more rhomboidal outlines, or from *H. sapiens* that tend to exhibit squared symmetrical contours [17], [41], [42], [73].

The heavily built and highly divergent lingual root exhibited by the Hexian PA836 can also be found in the specimens of East Asian Early and mid-Middle Pleistocene [52], [71]. In contrast, the European Early Pleistocene specimens have lingual roots that barely diverge [75]. The roots of Atapuerca SH M^1^s may only diverge in the apical third, and the lingual roots tend to curve buccally [43]. The degree of divergence of the lingual root in recent modern human is variable, with some of them being as divergent as that of the Hexian tooth. However, in modern humans the lingual root is less robust and narrows gradually towards the tip.

### Upper second molar (M^2^)

As for the M^2^, the hypocone size of Hexian M^2^ assessed by ASUDAS fits within the variability observed in *H. ergaster* and East Asian Early and mid-Middle Pleistocene specimens. In these groups, the hypocone is reduced compared to *Australopithecus* and African early *Homo* [76], [77] but remains primitive compared to the strong reduction of some European Middle Pleistocene groups, Neanderthals, and *H. sapiens* [18], [43]. The expression of a transverse crest is a highly polymorphic feature and it is not taxonomically useful [18].

Regarding the crown outline, the relative expansion of the wide mesial cusps with regard to the distal ones provides a characteristic trapezoidal shape that is common in *Australopithecus*, early *Homo* specimens, and most Asian Early to Middle Pleistocene fossils [17], [20], [52], [76], and unlike European Middle Pleistocene hominins, Neanderthals, and *H. sapiens* where the hypocone is proportionally more reduced than the metacone and the outline is either rhomboidal or triangular [78].

Compared with the data from Olejniczak et al. [59] and Smith et al. [60] (Table 3), the AET of PA833 is close to those of Asian *H. erectus* and North African Middle Pleistocene hominins, but it could be also enclosed within the range of variation of recent modern humans. Besides, the AET of PA833 is larger than that of European Middle Pleistocene *Homo* and Neanderthals. Regarding the RET, PA833 could also be included within the range of variation of recent modern humans, but it is larger than in any other taxonomic group (Table 3).

**Table 3:** Comparisons of AET (average enamel thickness) and RET (relative enamel thickness) between Hexian PA833 and other fossil hominins and recent modern humans (data in brackets indicate the maximum and minimum value when more than one specimen were involved).

	PA833^1^	AHE^2^	NAMP^2^	EMP^2^	Neanderthal^2^	Neanderthal^3^	EMH^2^	RMH^2^	RMH^3^
Number	1	1	1	1	6	6	1	29	25
AET (mm)	1.51	1.48	1.42	1.20	1.17 (1.09–1.29)	1.20	1.31	1.40 (1.13–1.84)	1.40
RET	23.52	19.42	18.81	17.13	17.06 (14.9–19.5)	18.12	19.8	21.40 (16.5–28.0)	21.59

AHE: Asian *H. erectus*, NAMP: North African Middle Pleistocene, EMP: European Middle Pleistocene, EMH: Early Modern Human, RMH: Recent Modern Human. 1, this study, 2, Smith et al. [60], 3, Olejniczak et al. [59].

### Lower second molar (M_2_)

The relative BL expansion of the M_2_s provides a characteristic broad and asymmetric contour to the Hexian teeth (particularly pronounced in PA833) that differs from the more symmetrical and elongated outlines found in African Pleistocene specimens such as KNWT-15000, KNMER-992, and KNMER-820 (see also Metric comparison).

The occlusal surfaces of the Hexian M_2_s are complex due to the development of accessory cusps, expression of deflecting wrinkle, and other features like secondary grooves or ridges. The co-expression of these accessory structures is commonly observed in *Australopithecus*, African Early Pleistocene *Homo*, East Asian Early and mid-Middle Pleistocene (but see the Bapang material reported by Zanolli [20]), and European Early Pleistocene fossils [15], [25], [35], [36], [52], [65], [66], [75], [79]–[81] and tend to reduce its frequency in European Middle Pleistocene hominins, Neanderthals, and modern humans [18], [43]. Occurrence of a Cusp 7 can be found in variable percentages in several hominin groups, being particularly high in European Middle Pleistocene groups and *H. neanderthalensis* [18], [43]. The occlusal grooves are arranged in a “Y” type, which is the primitive pattern for *Homo* and thus, not particularly informative in this case [14], [43]. The newly reported Sangiran M_2_s from the Kabuh (Bapang) Formation [20] are characterized by surprisingly derived features such as simplified occlusal surfaces, absence of hypoconulid, and a non-Y pattern of occlusal groove arrangement. Thus, the Hexian teeth would be remarkably more primitive than these specimens despite being younger.

**Figure 14:**
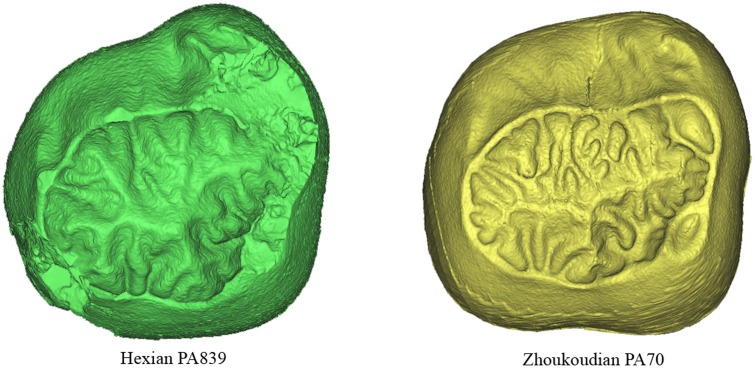
Comparison of the dentine surfaces of PA839 from Hexian (left) and PA70 from Zhoukoudian Locality 1 (right).

In parallel to the complexity assessed at the OES, the EDJ surfaces of the Hexian lower molars are profusely crenulated, more than they are usually found in Middle Pleistocene populations, Neanderthals, and *H. sapiens* [82]–[85]. This wrinkled EDJ surface could be a particularity of the Asian Pleistocene fossils as it has been also found in other Asian hominins such as Zhoukoudian PA70 (unpublished data and Fig. 14) although more data on Early Pleistocene fossils in general and on Asian hominins in particular is necessary to support this assessment. As shown in Fig. 14, the similarity between Hexian PA839 and Zhoukoudian PA70 is also notable in the strong lateral expansion of the buccal surface at the level of the protoconid and the complexity of the EDJ surface.

**Figure 15:**
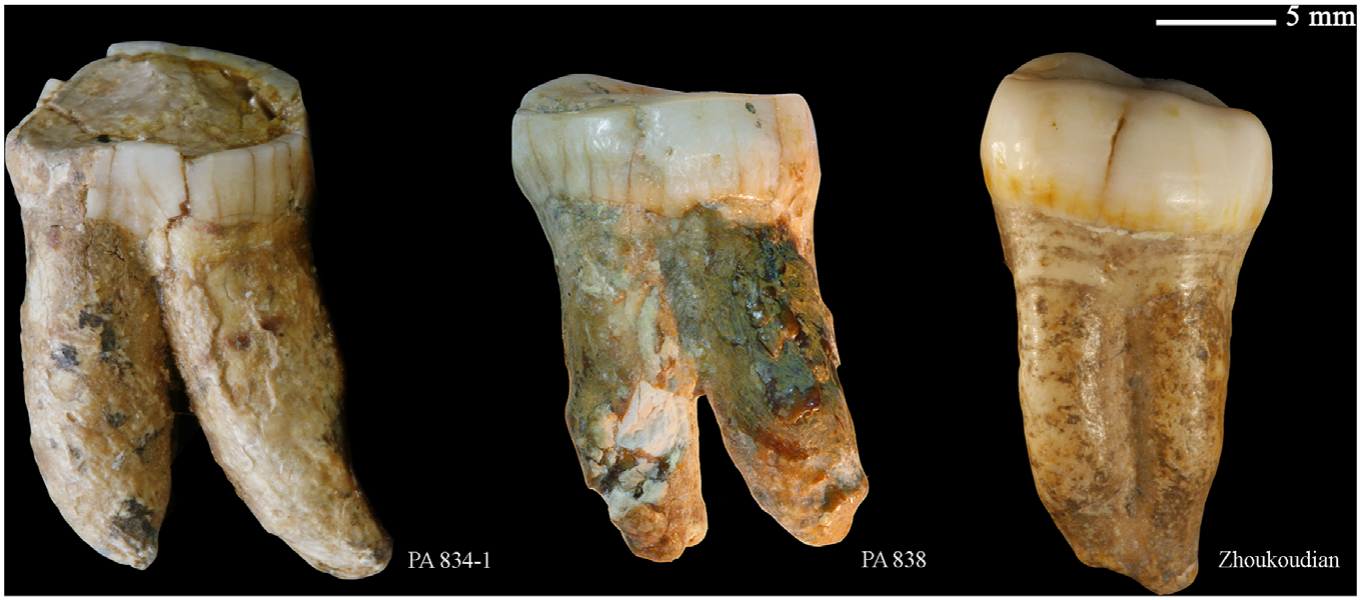
Buccal views of a sample of lower second molars from the East Asian mid-Middle Pleistocene period (From left to right: Hexian PA834-1, Hexian PA838, Zhoukoudian PA70).

In contrast, the roots of Hexian M_2_s roots are robust, with high bifurcating positions that are clearly different from the coalescing roots of Zhoukoudian Locality 1 (Fig. 15). The type of radicals that do not reduce their breadth until the apical fourth are typical of Asian Early Pleistocene specimens like those from Sangiran and Jianshi [1], [71]. Taurodont M_2_s have been reported on specimens of Zhoukoudian Locality 1 and especially Neanderthals [52], [86]. However, this feature [87] is not observed on the Hexian M_2_s.

### Lower third molar (M_3_)

The hypoconulid of Hexian M_3_ is well developed and buccally displaced as it is typical of *Australopithecus* and African and Asian Early and Middle Pleistocene hominins [15], [35], [36], [52], [65], [66] and in contrast to more reduced and central hypoconulids in European Pleistocene populations and modern humans [43].

The expression of a cusp 6 in M_3_ is more common in *Australopithecus* and African and Asian Early and Middle Pleistocene *Homo* than in European Pleistocene specimens and modern humans [15], [35], [36], [43], [52], [65], [66]. The middle trigonid crest is absent in the Hexian M_3_, and can be only found in low percentages in *Australopithecus*, African early *Homo*, *H. ergaster*, modern humans, and some specimens from Zhoukoudian Locality 1 [18], [35], [36], [43], [52], [65]. In contrast, in fossil hominins from East Asian Early Pleistocene and European Early and Middle Pleistocene, and particularly Neanderthals, the occurrence of this feature is relatively high [15], [43], [66].

An “X” groove type displayed by the Hexian M_3_ can be found on a few specimens of *Australopithecus* (30%), *H. ergaster* (KNM-ER 806, 1812), Zhoukoudian Locality 1 (ZKD131’) and 2 out of 3 M_3_s from the Sangiran (Kabuh) sample described by Zanolli [20] if their classification as M_3_s is confirmed, but it is not a common feature until the origin of the European Middle Pleistocene populations, Neanderthals, and *H. sapiens* [18], [43].

The Hexian M_3_ has both mesial and distal roots which are robust and well separated from the cervical region. This morphology is primitive and different from the more advanced morphologies found in the roughly contemporaneous fossils from Zhoukoudian, where roots tend to coalesce [52]. In addition, the fact that the roots do not narrow in the buccolingual direction until the tip is a typical feature of Early Pleistocene fossils from Asia like Sangiran [71]. Unlike Neanderthal samples [86] and one M_3_ from Zhoukoudian Locality 1 [52] the Hexian M_3_ does not present taurodontism.

### Upper central incisor (I^1^)

The Hexian I^1^ (PA 835) is characterized by pronounced shovel shape, a primitive feature that can be expressed in different degrees in *Australopithecus* and African early *Homo* [35], but becomes more pronounced and frequent in Pleistocene Eurasian populations, particularly Neanderthals [88]. With a similar distribution, convexity of the labial surface in I^1^s tends to be weak in African Pliocene and Pleistocene fossils and *H. sapiens* [14], [35], [36], [65], increasing its grade in Eurasian Pleistocene populations, and achieving the highest degrees of expression in European Middle Pleistocene groups and Neanderthals [43]. The labial convexity of Hexian I^1^ is less pronounced than in Zhoukoudian Locality 1, but within the variation of Indonesian Early Pleistocene hominins [52], [66].

**Figure 16:**
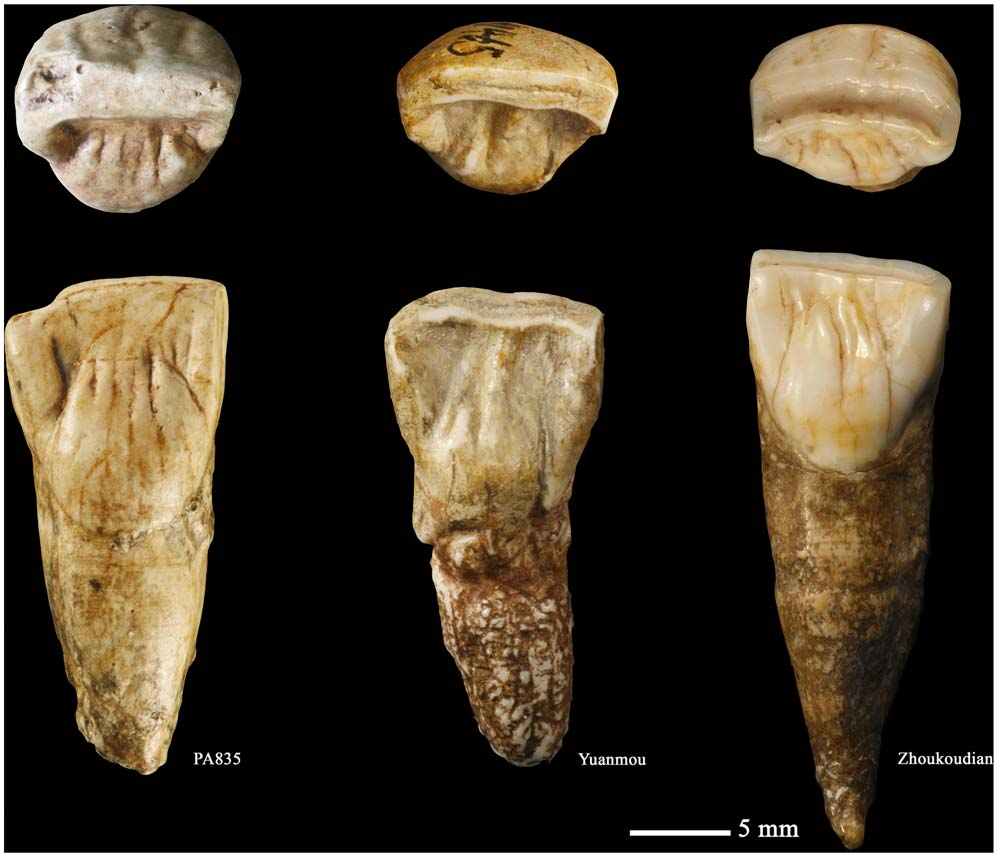
Occlusal and lingual views of three upper central incisors from Hexian (left), Yuanmou (middle), and Zhoukoudian (right).

The lingual surface of Hexian I^1^ displays a basal eminence and several finger-like projections, which should be treated as separate traits since their expression is not necessarily correlated [14], [18], [43]. A round and elevated basal eminence like that of Hexian I^1^ is typical of specimens from East Asian Early and mid-Middle Pleistocene fossils, European Middle Pleistocene groups, and Neanderthals, although not exclusive to them [18], [43]. Regarding the finger-like projections [52], this feature could represent a primitive conformation since early and recent modern humans tend to reduce their number [18], [43]. In this feature, the Hexian I^1^ would be more similar to *H. ergaster* and Zhoukoudian Locality 1 specimens (Fig. 16) and different from Indonesian Early Pleistocene fossil hominins due to the lack of finger-like projections in this group [66]. However, some Indonesian teeth express a spine-like structure that occupies a central position and can even reach the incisal edge to form a lingual central ridge [18]. This spine-like feature can be identified in specimens such as KNM ER-803, but mostly in East Asia Early and Mid-Middle Pleistocene specimens (S7–85, S7–86, Yuanmou, ZKD PA66).

Finally, a distinctive feature of Hexian I^1^ is the wrinkled labial surface of the tooth. The expression of several longitudinal ridges along this surface could correspond with the expression of a “cingulum” or buttressing structure in an upper incisor, an unusual trait that we have only identified so far in some other East Asia mid-Middle Pleistocene specimens such as PA66 from Zhoukoudian Locality 1 (Fig. 16).

## Metric Comparison

The crown metric data of Hexian and the comparative samples used in the present study are listed in S5 Table in S1 Text. We also provide the bivariate plots of the crown MD and BL diameters for all the individual specimens of the Hexian and the comparative samples (S1, S2, S3, S4, S5, and S6 Figs. in S1 Text).

**I^1^:** The MD diameter of PA835 exceeds the MD dimension of all the studied teeth except for a few specimens from the *Australopithecus* and the early *Homo* groups. In the BL dimension, PA 835 also falls at the upper limit of the range of variation of all the groups, and it is clearly larger than most specimens of *H. ergaster*, East Asian Early and mid-Middle Pleistocene groups. In both the MD and the BL dimensions, PA 835 is close to KNM WT-15000, which is also a large incisor.

**P^3^:** The MD diameter of PA 832 is larger than those of *H. ergaster*, European Early Pleistocene hominins, East Asian Late Middle Pleistocene hominins, and modern humans. It is also larger than almost all European Middle Pleistocene hominins and Neanderthals. Regarding the MD value, PA 832 distributes at the upper limit of the range of variation of East Asian Early and mid-Middle Pleistocene hominins, European Middle Pleistocene hominins, and Neanderthals. The BL of PA 832 is larger than those of all the studied groups except for a few *Australopithecus* and early *Homo* specimens.

**M^1^:** The MD diameter of PA 836 is larger than most specimens of the East Asian Middle Pleistocene, European Middle Pleistocene, Neanderthals, and early modern humans. In this value, PA 836 falls in the middle of the distribution area of *H. ergaster* and East Asian Early Pleistocene hominins. In the BL dimension, PA 832 falls at the upper limit of the distribution areas of East Asian Early and mid-Middle Pleistocene hominins, European Middle Pleistocene hominins, Neanderthals, and early modern human. The BL dimension of PA 836 exceeds those of all the *H. ergaster* and European Early Pleistocene hominins studied here.

**M^2^:** The MD dimensions of Hexian M^2^s exceed those of all the East Asian Middle Pleistocene specimens, and distribute at the upper limit of the range of variation of European Early and Middle Pleistocene hominins, Neanderthals, and early modern humans. In the MD dimension, the Hexian M^2^s fall within the distribution area of *H. ergaster* and East Asian Early Pleistocene hominins. The BL diameters are also large and fall at the upper limit or out of the range of variation of East Asian mid-Middle Pleistocene, European Early and Middle Pleistocene, Neanderthals, and modern humans.

**M_2_:** In the MD dimension, the Hexian M_2_s fall within the distribution area of *H. ergaster* and East Asian Early Pleistocene hominins, but they are larger than the East Asian mid-Middle Pleistocene specimens, European Early Pleistocene hominins, and modern humans. The MD diameter of Hexian M_2_s is only smaller than a few European Middle Pleistocene specimens and Neanderthals. In the BL dimension, the Hexian M_2_s exceeds all the East Asian mid-Middle Pleistocene hominins, Neanderthals, and modern humans. The BL diameter of Hexian M_2_s falls at the upper limit of the distributions of *H. ergaster* and the East Asian Early Pleistocene hominins.

**M_3_:** In the MD diameter, PA834-2 falls in the range of variation of *H. ergaster* and the East Asian Early Pleistocene hominins, and it is larger than all the East Asian mid-Middle Pleistocene specimens, European Early and Middle Pleistocene specimens, Neanderthals, and modern humans. The BL of PA834-2 is only smaller than those of the *Australopithecus* and early *Homo* groups.

In sum, the Hexian teeth present large dimensions that usually fall close to the lower limit of the variation of African Pliocene/Early Pleistocene specimens despite Hexian’s younger age. Metrically, the Hexian teeth are closer to the Early Pleistocene specimens from Indonesia than to assemblages with a closer chronology such as those from Zhoukoudian, especially the M^2^, M_2_, and M_3_. The dimensions of Hexian I^1^ are very similar to those of KNM WT-15000. However, and unlike *H. ergaster*, upper and lower molars of Hexian teeth are characterized by relatively broader crown outlines as it seems typical of Asian *H. erectus*.

## Discussion and Conclusions

In this study, the metric and morphological features of ten Middle Pleistocene hominin teeth from Hexian, China, were described and compared with several hominin teeth recovered from Africa, Asia, and Europe. As a result, it is possible to make some inferences regarding the similarities/differences of the Hexian hominins compared to the European and African record in general and within the Asian hominin record in particular.

The Hexian teeth were found to be relatively primitive within the genus *Homo* from both metric and morphological aspects. Metrically, they are large and fall in the upper limit of variation of the genus *Homo.* Hexian hominins tend to show relatively broad molar crowns, particularly at the level of the mesial cusps, which provides them with a characteristic primitive outline that is even more pronounced than in most of the Early and Middle Pleistocene fossil from Asia. Morphologically, the primitive status of Hexian dental crowns is shown by the expression of buttressing and accessory structures as well as the number, degree of divergence, and morphology of the roots. To be specific, the buttressing structures refer to the expression of a finger-like tuberculum dentale on the I^1^’s lingual face, and vertical grooves on the P^3^’s and I^1^ buccal/labial surfaces that delimit thickened mesial and distal ridges on this view. These features could have acted like buttressing systems or *mass-additive* traits [89], providing the tooth with a conspicuous morphological robusticity. Regarding the accessory structures, we find several secondary grooves that define multiple accessory ridges at the mesial marginal ridges of upper molars, the expression of deflecting wrinkles, accessory cusps like the C6, and a characteristic groove that crosses the protocone in the M^2^s. The latter seems to be a quite common feature in other Asian hominins from Zhoukoudian (e.g., ZKD41, 105) and Sangiran (e.g., S4 and S7–3). The presence of these accessory structures make the occlusal surface complicated in both the enamel and the dentine surfaces. In particular, the dentine surface is profusely crenulated with bifurcated essential crests and several ridges.

The primitive morphological pattern exhibited by the Hexian specimens distinguishes them from the European Middle Pleistocene hominins, Neanderthals, and modern humans. There are a series of dental studies pointing to some features that become the typical condition of the Neanderthal lineage because of their high degrees and frequencies of expression [40]–[43], [86], [90], [91], such as the high and continuous middle trigonid crest of lower molars, the pronouncedly buccal convexity of upper incisors, the skewed crown outline of M^1^ due to the reduced metacone and protruding hypocone, and taurodontism in molars. Some of these traits can be already found in *H. antecessor* [42], indicating that they are not Neanderthal apomorphies but features that appeared early in the evolution of the genus *Homo* and became typical of *H. neanderthalensis* because of their combination and frequent expression. Until now, the typical Neanderthal dental pattern in both the degree and combination of morphological features, has not been detected in East Asian Middle Pleistocene fossils [17], [19] and it is absent in the Hexian collection. As one of the earliest inhabitants of Europe, *H. antecessor* is similar to Hexian in the expression of some primitive traits like the extensive enamel crenulations, the development of secondary grooves on the occlusal surface of posterior teeth, and the expression of deflecting wrinkle [75]. However, some of the buttressing features such as the vertical grooves on the buccal surface of the upper premolars would be more pronounced in the Hexian specimens. Indeed, the fact that the Asian fossils do not share with *H. antecessor* and the Neanderthal lineage some of the dental features mentioned above, would be pointing to an early divergence of the Asian and the European Early Pleistocene populations.

Metrically and morphologically, the primitive traits of Hexian teeth make them similar to members of the *H. ergaster* taxon and to the East Asian Early and mid-Middle Pleistocene fossils. However, there are certain features of the Hexian teeth that are absent in *H. ergaster* and are more typical of Asian *H. erectus* such as the expression of pronounced vertical grooves on the labial surfaces of the Hexian P^3 ^s and I^1^, and the relative BL expansion of the lateral walls of the mesial cusps with regard to the distal ones in upper and lower molars. In addition, this study has also found that the relative enamel thickness of Hexian M^2^ was higher than the mean of any of the comparative groups (Table 3) although it could be enclosed within the range of variation of recent modern humans. If we assume that having relatively thin enamel is a derived feature in the Neanderthal lineage, as tentatively suggested by Smith et al. [60], the Hexian teeth would be also primitive for this feature, being thicker than the Zhoukoudian specimen analyzed by Smith et al. [60]. Nevertheless, more data on larger archaic *Homo* samples are needed to understand the polarity of the dental tissue proportions.

With regard to the Asian hominin record, previous observations on other Hexian remains beside teeth, like the skull, suggested stronger affinities with the Indonesian specimens than with those from Zhoukoudian Locality 1 [2], [3], [92]. Instead, some researchers in support of the regional continuity of the morphologies of the Chinese fossil record [5], [7], [8] were inclined to emphasize the similarities of the Hexian and Zhoukoudian V skulls, particularly in relation with some advanced features that, according to them, were not detected on the skulls recovered from the lower layers of Zhoukoudian Locality 1. Nevertheless, recent multivariate analyses on cranial metrical variation in East Asia during the Early and the mid-Middle Pleistocene periods pointed out that the Hexian skull was clearly differentiated from the specimens found in Zhoukoudian Locality 1, including skull V [9]. In the present study, we have also revealed several metrical and morphological differences when comparing the teeth from Hexian and Zhoukoudian Locality 1. Comparatively, the I^1^, M^2^, M_2_, and M_3_ from Hexian are generally much larger than those from Zhoukoudian Locality 1. Besides, the medium-sized hypocone of M^1^s and the taurodontism of some Zhoukoudian molars are also different from the Hexian dental sample [18], [52].

In addition to the crown features, the three independent and widely divergent roots of the P^3^ and the strong mesial and distal roots of the M_2_ and M_3_ are more primitive than those from Zhoukoudian Locality 1. Aside from *Australopithecus* and early *Homo,* a three-rooted P^3^ has been only identified in the East Asian Early Pleistocene (Pucangan) hominins [36], [65], [66], [69], but not in the Bapang Formation or the Zhoukoudian collection [52]. Moreover, the roots of Hexian teeth present rounded sections that barely narrow until the tip, a feature that seems typical in Asian Early Pleistocene teeth. This type of strong molar roots that are thick throughout their entire length were already reported in other Asian specimens such as Sangiran 21, Sangiran 17, Sb 8103, and Ng 8503 [15], [71]. According to Kaifu [71] this condition would be also present in other archaic hominins like those from Zhoukoudian, although our own observations point to relatively narrower and less divergent (and even coalescing roots) in this population despite their later chronology. Thus, compared to other Asian groups, the Hexian hominins preserved the primitive root conformation, apart from generally larger crown dimensions that would not follow the dental reduction processes ascertained in other Early and Middle Pleistocene assemblages from Asia [14], [71].

The comparison with other fossils from the Late Middle Pleistocene period in East Asia, such as Chaoxian [17] and Panxian Dadong [19] also reveals morphological differences among the hominins of this period and region. Regarding the of P^3^, M^1^ and M^2^, the Chaoxian teeth are similar to those from Hexian, and consistent with the morphological patterns ascertained in the Early and early Middle Pleistocene periods in China and Indonesia. However, the Hexian and Chaoxian teeth differ in their crown sizes, with the former showing larger crowns. In contrast, the specimens from Panxian Dadong are both metrically and morphologically more derived than those from Hexian and Chaoxian, displaying some features that fall within the range of variation of modern humans [19].

The recent work by Zanolli [20] has further expanded the morphological and metric variability of the specimens from the Sangiran Dome. Although the precise chronology of the Sangiran Kabuh Formation remains debated [47], [49], [50], we agree with Zanolli [20] that the Pucangan Formation and “Grenzbank zone” (lowest part of Kabuh) molars are in general more primitive than some of the later Kabuh Formation specimens. This is particularly true for the samples recently described by Zanolli [20] and in contrast with those discussed at Grine and Franzen [66]. Summarizing we could state that the primitive status of the Hexian teeth is closer to that of the Indonesian Early Pleistocene fossils than to Zhoukoudian hominins, although the dimensions and certain features of the Hexian remains are also outside of the variation of the Javanese specimens. Specifically, the crowns sizes of the Hexian I^1^ and P^3^ are larger than those from the Indonesian Early Pleistocene. In addition, the finger-like tuberculum dentale of the Hexian I^1^ is not found in the Sangiran assemblage. Therefore, Hexian fossils would expand the metrical and morphological variation known for the East Asian hominins in the mid-Middle Pleistocene.

Summarizing, we could state that the Hexian teeth are more primitive than the Zhoukoudian teeth and comparable in some features to those of *H. ergaster* and the earlier specimens from Indonesia. The latest and more reliable chronological estimation of Hexian provided an age of ca. 412 ka ([32], see Hexian site section for a discussion). Regarding the site of Zhoukoudian, the latest geochronological assessments point to an early to mid-Middle Pleistocene date for these hominins [93]–[96], making Hexian roughly contemporaneous or likely older than Zhoukoudian. Thus, the relative younger geological age of the Hexian hominins in comparison with the African and Javanese Early Pleistocene fossils as well as the Zhoukoudian hominins would contrast with the more primitive metric and morphological features of the former.

Our results point to certain features common to many Asian Early and Middle Pleistocene fossils that could be part of a *H. erectus* dental bauplan such as the robusticity and rounded section of the roots throughout its whole length, the shape of upper and lower molars with the lateral expansion of the mesial cusps, and possibly the highly crenulated dentine surfaces. However, future studies on more dental Asian samples are needed to confirm this pattern. Interestingly, and despite some common traits among these samples, our study suggests that the primitive-derived gradients of the Asian hominin samples cannot be satisfactorily fitted along a chronological sequence, possibly suggesting complex evolutionary scenarios with the coexistence and/or survival of different lineages in Eurasia. The Hexian materials could represent the persistence in time of a *H. erectus* group that would have retained primitive features that were lost in other Asian populations such as Zhoukoudian or Panxian Dadong. Several researchers have already pointed out an evolutionary scenario in Asia that envisages persistence and survival of certain hominin groups as a result of the evolution in biogeographical isolation [88], [97]–[100]. A scenario of discontinuity and regional evolution has been previously suggested for the Javanese and the Asian continental populations [20], [71], [97], [98]. Despite the dental morphological differences found between the chronologically younger and older specimens recovered from the Sangiran sequence, the variability of the assemblage remains relatively stable, pointing to certain degree of morphological stasis in this region during a relatively long time period [15], [16], [71]. Similarly, it has been suggested that the hominins recovered from Zhoukoudian Locality 1 have remained relatively unchanged until the late Middle Pleistocene [18], [101]–[105] despite covering a time range of approximately 300 ka. In this context, and except for the metric variables, the Zhoukoudian fossils could not be interpreted as “intermediate” between the younger and the older fossils of the Sangiran sequence [97], supporting regional variation and evolution. Regarding the Hexian sample, and in support of a scenario of isolation in continental Asia, recent studies [106], [107] suggest that the location and varying extent of deserts are likely to have had major impacts upon hominin populations across Asia. The loess-palaeosol sequences of China and Central Asia provide among the best proxy records of desert expansion and contraction [108]. In particular, in North and Central China, as well as in Central Asia, “cold dry periods were associated to reduced vegetational cover and increased dust deposition, blown in from neighboring deserts, often in violent dust storms” [106]. Deserts have repeatedly expanded and contracted during the Pleistocene, with some becoming more extensive during the Middle Pleistocene, impeding or preventing hominin dispersals across Asia, particularly from ca. 500 ka onwards. Indeed, the recent analysis of the pattern of hominin settlement in the Nihewan Basin and adjacent Loess Plateau suggest that hominin occupation was likely intermittent and linked to favorable warm periods [107]. Future studies should explore whether the morphological variability of the Asian populations can be explained by a “source-sink” model, as it was recently proposed for Western Europe [109], [110]. As suggested by Dennell [106], [107] semi-arid regions probably contained the source populations that could expand into adjoining arid regions when either population levels allowed expansion and/or when climatic conditions were conducive to expansion [106]. Sink populations would be those that required recruitment from source areas to remain viable. The climatic instability and environmental change in Asia might have favored a pattern of fragmentation and isolation leading to the high morphological variability of the Asian hominin record and the possibility of persistence of primitive hominin lineages throughout time. However, a wider study integrating the morphological and the biogegraphical information is necessary in order to test this model in Asia, in general, and in Central and Southern China in particular.

In sum, our analysis and comparison of the Hexian human fossil assemblage has contributed to a better understanding of the morphological variability of the *H. erectus* species, but more detailed studies of old and new hominin assemblages from Asia are necessary. The general inclusion of all the human fossils from China and Indonesia into *H. erectus* may be indeed oversimplifying the evolutionary course of all the human populations that inhabited for about two million years in an area that is larger than 10 million square kilometers. Apart from the identification of certain common features that fit within the variability of the Early and Middle Pleistocene populations of Java and continental Asia, our study highlights the relatively primitive metric and morphological dental features of the Hexian hominins if we compare them to other contemporaneous or even younger populations of mainland Asia.

## Supporting Information

S1 Text **Supporting files. S1 Fig.** Bivariate plots of the crown sizes of I^1^s of the Hexian and the comparative samples (convex hulls were used to graphically highlight the distribution area of *H. erectus* sensu lato). **S2 Fig.** Bivariate plots of the crown sizes of P^3^s of the Hexian and the comparative samples (convex hulls were used to graphically highlight the distribution area of *H. erectus* sensu lato). **S3 Fig.** Bivariate plots of the crown sizes of M^1^s of the Hexian and the comparative samples (convex hulls were used to graphically highlight the distribution area of *H. erectus* sensu lato). **S4 Fig.** Bivariate plots of the crown sizes of M^2^s of the Hexian and the comparative samples (convex hulls were used to graphically highlight the distribution area of *H. erectus* sensu lato). **S5 Fig.** Bivariate plots of the crown sizes of M_2_s of the Hexian and the comparative samples (convex hulls were used to graphically highlight the distribution area of *H. erectus* sensu lato). **S6 Fig.** Bivariate plots of the crown sizes of M_3_s of the Hexian and the comparative samples (convex hulls were used to graphically highlight the distribution area of *H. erectus* sensu lato). **S1 Table**, Chronologies of the Hexian hominins. **S2 Table**, Specimens used in the morphological comparisons. **S3 Table**, Specimens used in the linear metric comparisons. **S4 Table**, Morphological comparisons of the Hexian hominins and other members of *H. erectus* sensu lato. **S5 Table**, Mean and Std. Deviation of the crown size of the Hexian teeth and comparative samples.
(DOCX)
