# Supplementary material for: Middle Pleistocene Hominin Teeth from Longtan Cave, Hexian, China
Source: PLoS One. 2014 Dec 31;9(12):e114265. doi: 10.1371/journal.pone.0114265 (PMC4281145; doi:10.1371/journal.pone.0114265)
Supplement: S1 Text — Supporting files. S1 Fig. Bivariate plots of the crown sizes of I1s of the Hexian and the comparative samples (convex hulls were used to graphically highlight the distribution area of H. erectus sensu lato). S2 Fig. Bivariate plots of the crown sizes of P3s of the Hexian and the comparative samples (convex hulls were used to graphically highlight the distribution area of H. erectus sensu lato). S3 Fig. Bivariate plots of the crown sizes of M1s of the Hexian and the comparative samples (convex hulls were used to graphically highlight the distribution area of H. erectus sensu lato). S4 Fig. Bivariate plots of the crown sizes of M2s of the Hexian and the comparative samples (convex hulls were used to graphically highlight the distribution area of H. erectus sensu lato). S5 Fig. Bivariate plots of the crown sizes of M2s of the Hexian and the comparative samples (convex hulls were used to graphically highlight the distribution area of H. erectus sensu lato). S6 Fig. Bivariate plots of the crown sizes of M3s of the Hexian and the comparative samples (convex hulls were used to graphically highlight the distribution area of H. erectus sensu lato). S1 Table, Chronologies of the Hexian hominins. S2 Table, Specimens used in the morphological comparisons. S3 Table, Specimens used in the linear metric comparisons. S4 Table, Morphological comparisons of the Hexian hominins and other members of H. erectus sensu lato. S5 Table, Mean and Std. Deviation of the crown size of the Hexian teeth and comparative samples. (DOCX) [file pone.0114265.s001.docx]

Text S1. Middle Pleistocene hominin teeth from Hexian, China

**Table S1**. Chronologies of the Hexian hominins.

| Samples | Methods | Chronology | Reference |
| --- | --- | --- | --- |
| Faunal composition | --- | Corresponds to the fifth layer of the Zhoukoudian Locality 1(i.e. 320-330 ka) | [1, 2] |
| Quartz | TL | 184-195ka | [3] |
| Mammal teeth | U-series | 150-270ka | [4] |
| Mammal teeth | ESR | 300ka | [5-7] |
| Mammal teeth | ESR+ U-series | 412±25ka | [8] |

**Table S2.** Specimens used in the morphological comparisons.

| Geography and Chronology | Specimens |
| --- | --- |
| **Africa** |  |
| Pliocene (*Australopithecus*) (n=129) | AL128-23, 145-35, 188-1, 199-1, 200-1, 266-1, 277-1, 288-LI, 333-86, 333w-1, 333w-32, 333w-48, 333w-57, 333w-60, 400-1; LH3, 4, 5, 6, 11, 15, 17, 21, 23; MLD2, 11, 19, 23, 28, 30, 43, 44, 45, 128; Stw3, 14, 19, 47, 59, 61, 72, 73, 75l, 90, 96, 107, 109, 120, 151, 183a, 188, 192a, 196, 202, 204, 206, 212, 213, 234, 235, 237, 252, 269, 278, 281, 283, 285, 286, 295, 306, 308, 312, 322, 327, 353, 384, 386, 397, 402, 404, 412, 424, 447, 450, 487, 498, 519, 520, 530, 531, 534, 540, 551, 555, 560d, 586, 632 |
| Late Pliocene and Early Pleistocene (Early *Homo*) (n=44) | KNM ER-808, 809, 1462, 1470, 1480, 1482, 1506, 1590, 1802, 1805, 1813, 3734; OH4, 6, 7, 13, 16, 21, 24, 27, 37, 39, 44, 45, Stw53 |
| Late Pliocene and Early Pleistocene (*H. ergaster*) (n=20)  North African Middle Pleistocene (n=10) | KNM ER-803, 806, 807, 992, 1808, 1812, 3733; KNM WT-15000; Stw80  Rabat; Ternifine 1, 2, 3, 10 |
| Holocene (Recent modern human) (n=130) | Mesolithic North African Sample (Tebessa, Aïn Meterchem, Gambetta, Aïn Dokkara, Taforalt |
| **East Asia** |  |
| Early Pleistocene (n=38) | S1b, 4, 5, 6b, 7-1, 7-3, 7-8, 7-9, 7-10, 7-13, 7-27, 7-31, 7-34, 7-37, 7-38, 7-40, 7-48, 7-49, 7-53, 7-58, 7-64, 7-65, 7-68, 7-85, 7-86, 7-89, 9, 17; Yuanmou |
| Mid-Middle Pleistocene (n=33)  Late Middle Pleistocene (n=4) | ZKD1, 3, 4, 19, 32, 33, 40, 41, 42, 44, 45, 50, 52, 77, 95, 104, 105, 106, 107, 108, 110, 111, 140’, 144’, 145’, PA66  Chaoxian; Panxian Dadong |
| Late Pleistocene (Early modern human) (n=11) | Huanglong Cave, Liujiang, Xintai, Zhiren Cave, Zhoukoudian (ZKD) Upper Cave |
| Holocene (Recent modern human) (n=501) | Contemporary modern sample from China (Henan Province and Hubei Province) |
| **West Asia** |  |
| Early Pleistocene (n=16) | Dmanisi (D211, D2282, 2375, 2600, 2700, 2710, D2736, D3672) |
| Late Pleistocene (Early modern human) (n=28) | Qafzeh 2, 4, 5, 6, 7, 9, 11 |
| **Europe** |  |
| Early Pleistocene (n=15) | Atapuerca Gran Dolina (ATD6-5, ATD6-7, ATD6-10, ATD6-11, ATD6-12, ATD6-13, ATD6-69, ATD6-103, ATD6-113) |
| Middle Pleistocene (n=205) | Arago (6, 9, 10, 13, 14, 21, 31, 32, 36, 54, 61, 68, 69, 87, 89, 106, 108, 109, 111); Atapuerca Sima de los Huesos (AT-1, AT-8, AT-11, AT-12, AT-13, AT-15, AT-16, AT-20, AT-24, AT-26, AT-27, AT-30, AT-41, AT-42, AT-46, AT-54, AT-75, AT-138, AT-139, AT-143, AT-146, AT-165, AT-169, AT-170, AT-196, AT-197, AT-198, AT-199, AT-222, AT-270, AT-271, AT-273, AT-278, AT-280, AT-284, AT-300, AT-405, AT-406, AT-407, AT-421, AT-505, AT-553, AT-554, AT-557, AT-560, AT-587, AT-588, AT-589, AT-598, AT-599, AT-605, AT-607, AT-700, AT-767, AT-772, AT-792, AT-793, AT-810, AT-811, AT-812, AT-813, AT-814, AT-815, AT-817, AT-821, AT-822, AT-823, AT-824, AT-827, AT-888, AT-941, AT-942, AT-944, AT-946, AT-950, AT-953, AT-954, AT-959, AT-960, AT-1100, AT-1130, AT-1143, AT-1463, AT-1468, AT-1473, AT-1632, AT-1752, AT-1756, AT-1761, AT-1943, AT-1944, AT-1945, AT-1957, AT-1958, AT-1959, AT-2036, AT-2071, AT-2076, AT-2175, AT-2179, AT-2193, AT-2270, AT-2271, AT-2272, AT-2273, AT-2277, AT-2385, AT-2389, AT-2395, AT-2396, AT-2399, AT-2438, AT-2752, AT-2758, AT-2760, AT-2763, AT-2764, AT-2770, AT-2777, AT-2782, AT-2786, AT-3176, AT-3177, AT-3178, AT-3179, AT-3182, AT-3185, AT-3186, AT-3193, AT-3194, AT-3424, AT-3885, AT-3889, AT-3890, AT-3943, AT-4147, AT-4156, AT-4317, AT-4319, AT-4320, AT-4322, AT-4323, AT- 4325, AT-4326, AT-4330, AT-4336, AT- 4624, AT-5611, AT-5615, AT-5804, AT-5838, AT-5899,); Mauer; Montmaurin; Pontnewydd (PN2, PN21, PN8, PN15) |
| Neanderthals (n=114) | Amud I; Arcy-sur-Cure (Bison, Hyène 1, Hyène 4, Hyène 5, Renne5, Renne 19, Renne21, Renne 39, Renne45); Breuil 3; Cabezo Gordo; El Sidrón (SDR012); Guattari (2,3); Hortus (H3, H4, H5, H13, H346, H352, H405, H549, H550, H695, H884, H977, H987, H1011); Krapina (Kra1, Kra6, Kra7, Kra9, Kra10, Kra38, Kra48, Kra53, Kra55, Kra58, Kra86, Kra92, Kra93, Kra94, Kra100, Kra101, Kra106, Kra107, Kra109, Kra110, Kra116, Kra123, Kra129, Kra132, Kra133, Kra134, Kra136, Kra155, Kra157, Kra165, Kra166, Kra169, Kra171, Kra172, Kra175, KraA, KraB, Kra MxB, KraC, KraD, Kra MxE, KraG, KraH, KraJ); Kulna; La Quina (5, 9); Le Moustier; Monsempron; Pech-de-l’Azé; Petit-Puymoyen Mx6; Pinilla del Valle (PIN10); Regourdou 1; Shanidar 2; St. Césaire; Tabun (TB1); Subalyuk 1; Vindija; Zafarraya |
| Late Pleistocene (Early modern human) (n=72) | Abri Pataud 1; Brassempouy (BR90, BR94); Dolní Vĕstonice (13, 14, 15, 31, 36.9, 37, 38); Grimaldi; Isturitz (105, 107, 115); L’Espugo; Les Rois (A, 31, R6, #54, R53 P, R50, R50#3, R51#4, R50#5, R51#29, R50#45, w/#); Mladeč; Pavlov (1, 2.1-2.2, 28); Saint Germain-La Riviere (11, 18, 21, SG-LR1, SG-LR2, SG-LR3, SG-LR B6, SG-LR12, SG-LR15, SG-LR16, SG-LR19); Trou Magrite |
| Holocene (Recent modern human) (n=638) | Hispanic-muslim medieval collection of San Nicolás, Murcia, Spain, Mesolithic French sample (Tévic and Hoëdic); Neotlithic French sample (Avize, Dolmens de Bretons, Caverne de L’Homme Mort, Orrouy) |

**Table S3.** Specimens used in the linear metric comparisons.

| Geography and Chronology | Specimens | Reference |
| --- | --- | --- |
| **Africa** |  |  |
| Pliocene (*Australopithecus*) (n=108) | AL128-23, 145-35, 188-1, 198-1, 199-1, 200-1, 266-1, 277-1, 288-LI, 333-86, 400-1, 333w-1, 333w-32, 333w-48, 333w-57, 333w-59, 333w-60; LH3, 4, 6, 11, 15, 17, 21, 23; Stw3, 14, 18, 47, 59, 61, 72, 73, 90, 91, 107, 109, 120, 134, 149, 151, 183a, 188, 192a, 212, 213, 234, 235, 237, 252, 269, 278, 281, 283, 286, 295, 306, 308, 312, 321,327, 353, 384, 386, 402, 404, 412, 424, 447, 450, 487, 498, 502, 519, 520, 530, 531, 534, 540, 551, 555, 560d | [0, 9] |
| Late Pliocene and Early Pleistocene (Early *Homo*) (n=50) | KNM ER-808, 1462, 1480, 1482, 1506, 1590, 1802, 1805, 1813, 3734; OH4, 6, 7, 13, 16, 21, 24, 27, 37, 39, 41, 44, 45; Stw53 | [0, 9, 10] |
| Late Pliocene and Early Pleistocene (*H. ergaster*) (n=19) | KNM ER-730, 803, 806, 992, 1808, 1812, 3733; KNM WT-15000 | [0, 11, 12] |
| North African Middle Pleistocene (n=18) | Sidi Abderrhaman 1; Ternifine 1, 2, 3, 10; Thomas 1, 3; Rabat 1 | [0, 13] |
| **East Asia** |  |  |
| Early Pleistocene (n=55) | Bpg 2001.4, S1b, 4, 5, 6b, 7-1, 7-3, 7-8, 7-9, 7-10, 7-27, 7-31, 7-32, 7-34, 7-35, 7-37, 7-38, 7-40, 7-53, 7-58, 7-64, 7-65, 7-78, 7-85, 7-86, 7-89, 8, 9, 15b, 16, 17, 21, 22, BK 7905, SB 8103, Ng 8503, NG92.1, NG92.4, NG9107.2, NG0802.3, PCG09_KII_Z:1.37, Tjg1993.05; Yuanmou | [14-22] |
| Mid-Middle Pleistocene (n=35)  Late Middle Pleistocene (n=4)  Late Pleistocene (Early modern human) (n=1) | ZKD1, 3, 4, 19, 32, 33, 39, 40, 41, 42, 44, 45, 50, 51, 52, 77, 78, 95, 105, 106, 110, 111, 114, 116, 117, 131’, 136’, 138’, 140’, 142’, 144’, 145’, PA66, 67, 70  Chaoxian; Panxian Dadong  Zhoukoudian (ZKD) Upper Cave | [23, 24]  [25, 26]  [0] |
| **West Asia** |  |  |
| Early Pleistocene (n=11) | Dmanisi (D211, D2282, D2375, D2600, D2700, D2736, D3672) | [27] |
| Late Pleistocene (Early modern human) (n=67) | Qafzeh 3, 3A, 4, 5, 6, 7, 8, 9, 10, 11, 12, 12A, B11-70, B11-UNN-1; Skhul 1, 2, 4, 5, 6, 7, 9A, 10 | [0] |
| **Europe** |  |  |
| Early Pleistocene (n=11) | Atapuerca Gran Dolina (ATD6-5, ATD6-7, ATD6-10, ATD6-12, ATD6-13, ATD 6-69, ATD 6-113) | [28, 29] |
| Middle Pleistocene (n=146) | Arago (1, 2, 7, 13, 21); Atapuerca Sima de los Huesos (AT-1-I, AT-8, AT-11-XIII, AT-12, AT-13-VII, AT-15-VIII, AT-16/139-VII, AT-20-XVII, AT-27/42-II, AT-30-XXVI, AT-41, AT-54, AT-75-VI, AT-138/26-XII, AT-143, AT-146, AT-165/814-XXVIII, AT-169-X, AT-170-XII, AT-196-XXIV, AT-197, AT-198/199, AT-222-XVI, AT-270/46-VII, AT-273/271-III, AT-278, AT-280, AT-405/589-XX, AT-406/587-XX, AT-407/821-XX, AT-505-XIX, AT-553/554-VII, AT-557/1761-XI, AT-560, AT-598/1468, AT-605-XXII, AT-607-XXIII, AT-700-XXI, AT-767/960-XVI, AT-700/3424-XXI, AT-767/959-XVI, AT-772-XXVII, AT-792-XXVII, AT-793-IV, AT-810/4326-IV, AT-811-IV, AT-812-XIX, AT-813-VIII, AT-822-XVII, AT-823-XVII, AT-824, AT-827/815-XIX, AT-888-XXI, AT-941/1752-XVIII, AT-942, AT-946/3890-XX, AT-950-XXVIII, AT-953/954-XX, AT-1100/817-III, AT-1130-V, AT-1143-2395-XVIII, AT-1463/24-VII, AT-1473, AT-1632-XXVI, AT-1943, AT-1944/772-XXVI, AT-1945/1959, AT-1957-VII, AT-1958, AT-2036/2399-XVIII, AT-2076/2071-XVIII, AT-2179/2175-XVIII, AT-2193-XV, AT-2270/1756-XXVI, AT-2271/2277-XVIII, AT-2272/284-XIV, AT-2273, AT-2396/2438-XXIV, AT-2385/2438-XXIV, AT-2389/1130-V, AT-2752/2786-XVI, AT-2760/599, AT-2763-XVI, AT-2764/2758-XVI, AT-2770/3178-IV, AT-2777, AT-2782/4156-XXII, AT-3176/792-XXVII, AT-3177-VIII, AT-3179/421-II, AT-3182, AT-3185, AT-3186, AT-3193, AT-3194, AT-3885, AT-3889-XXV, AT-3943-XXV, AT-4147/300-XII, AT-4317/944-XXII, AT-4319/588-XXII, AT-4320-XXII, AT-4322/4330-XII, AT-4323-XXVIII, AT-4325, AT-4336-XXVIII, AT-4624, AT-5611, AT-5615, AT-5838-XI, AT-5899-XI); Mauer; Montmaurin; Petralona; Steinheim | [0, 30] |
| Neanderthals (n=211) | Amud (1, 5); Arcy-sur-Cure (8, 9, H1, H2, 2931, S2); Chateauneuf 2; Ehringsdorf (6, 7, 8, G/7); Genay (Côte d'Or) 1; Gibralter 1; Hortus (3, 4, 5, 6, 7, 8, 10, 549, 550, 766); Krapina (6, 9, 39, 86, 92, 94, 97, 100, 107, 110, A/B, B/A, D/D, D, E, F/H, N/N, Md C, Md E, Md G, Md K, Md J, Md L, Md M, Mx C, Mx E, Mx G, Mx H, Mx J, Mx K, Mx L, Mx M, Mx O, Mx P, Mx Q); Kulna 1; La Chaise (8, 17, 18, 38); La Ferrassie 2; La Quina (5, 9, 18, 20); Le Moustier 1; Monsempron; Ochoz 1, 2; Pech de l'Azé; Petit Puymoyen (1, 3, 4); Regourdou 1; Saccopastore 1, 2; Sakajia; Shanidar (1, 2, 6); Spy (1, 2); St. Césaire 1; Subalyuk 1; Tabun (1, 1-1, 2, 2-1, 2-3, 3-2, B1, B4,); Vindija (206, 229, 231, 259, 290) | [0] |
| Late Pleistocene (Early modern human) (n=112) | Abri Pataud (1, 2, 4, 6); Combe Capelle 1; Cro-Magnon (2, 4, 5); Dolní Vĕstonice 3; Fontechevade 2; Isturitz (Series 6-7) (6B, 7B-1); Le Rois (INC-3, B R5#10, R50#3, R50#5, R50#31, R50#45, R54); Les Vachons 1; Mladeč (1, 2A, 5453, 1903 MX 1, 1904 MX 1, 1904 MX 2, 1903 Md 1, 1904 Md 5); Pavlov 1; Predmostí (1, 2, 3, 4, 5, 7, 9, 10, 14, 18, 22, 26, 27 ,476, 3070); Zlaty Kun 1 | [0] |
| Holocene (Recent modern human) (n=362) | Upper Paleolithic and Mesolithic European samples (Denmark; France; Germany; Portugal) | [31] |

*”0”: contributed by Wolpoff

**Table S4.** Morphological comparisons of the Hexian hominins and other members of *H. erectus* sensu lato.

|  |  | Hexian | *H. ergaster* | East Asian Early Pleistocene | East Asian mid-Middle Pleistocene |
| --- | --- | --- | --- | --- | --- |
| P^3^ | Transverse crest occurrence | 50% | Low | High | Low |
|  | Buccal vertical groove | None or pronounced | Faint to weak | Weak to pronounced | Weak to pronounced |
|  | Crown outline shape | Asymmetrical | Asymmetrical | Asymmetrical | Asymmetrical |
|  | Root number | 3 | 2 | 2 to 3 | 2 |
| M^1^ | Hypocone size | Large | Large | Medium to large | Medium to large |
|  | Crown outline shape | Asymmetrical trapezoidal with an oblique buccal contour | Rhomboidal | Asymmetrical trapezoidal with an oblique buccal contour | Asymmetrical trapezoidal with an oblique buccal contour |
|  | Root structure | Highly divergent | Highly divergent | Highly divergent | Highly divergent |
| M^2^ | Hypocone size | Medium | Medium to large | Medium to large | Large |
|  | Crown outline shape | trapezoidal | trapezoidal | trapezoidal | trapezoidal |
| M_2_ | Crown outline shape | Asymmetrical BL-expanded rounded | Symmetrical MD-elongated elliptical | Symmetrical MD-elongated elliptical or Asymmetrical BL-expanded rounded | Asymmetrical BL-expanded rounded |
|  | Occlusal surface complexity | high | high | high | high |
|  | Occlusal groove pattern | Y | Y | Y or + | Y or + |
|  | EDJ surface | Highly crenulated | --- | --- | Highly crenulated |
|  | Root structure | Highly bifurcated | Highly bifurcated | Highly bifurcated | From bifurcated to coalesced |
|  | Taurodontism | No | --- | --- | Yes |
| M_3_ | Occlusal groove pattern | X | Y or X | Y or X | Y or + |
|  | Root structure | Highly bifurcated | Highly bifurcated | Highly bifurcated | coalesced |
| I^1^ | Shovel shape | Pronounced | Moderate | Faint or moderate | Pronounced |
|  | Labial convexity | Moderate | Moderate | Faint to strong | Moderate |
|  | Numbers of finger-like projections | 5 | 2-5 | 0 | 2-5 |
|  | Buccal surface | Highly wrinkled | Relatively smooth | Relatively smooth | Highly wrinkled |

*The degrees of buccal vertical groove is scored gradiently as follows: 1) Smooth; 2) Faint or Depression; 3) Weakly developed; 4) Moderate; 5) Pronounced; 6) Strong, and slightly rolled. The grades of the other features in this table are scored as Turner et al. [32].

**Table S5.** Mean and Std. Deviation of the crown size of the Hexian teeth and comparative samples.

|  | I^1^ | | | | P^3^ | | | | M^1^ | | | |
| --- | --- | --- | --- | --- | --- | --- | --- | --- | --- | --- | --- | --- |
|  | MD | | BL | | MD | | BL | | MD | | BL | |
|  | Mean | SD | Mean | SD | Mean | SD | Mean | SD | Mean | SD | Mean | SD |
| Hexian | 11.70 | --- | 9.40 | --- | 9.13 | 0.18 | 13.29 | 0.16 | 12.30 | --- | 13.7 | --- |
| Pliocene (*Australopithecus*) | 10.76 | 1.10 | 8.45 | 0.53 | 9.39 | 0.48 | 13.00 | 0.91 | 12.79 | 1.09 | 13.80 | 1.03 |
| African Late Pliocene and Early Pleistocene (Early *Homo*) | 11.13 | 1.15 | 7.97 | 0.58 | 8.75 | 0.80 | 12.00 | 0.80 | 12.92 | 0.76 | 12.99 | 0.67 |
| African Late Pliocene and Early Pleistocene (*H. ergaster*) | 10.93 | 1.17 | 8.63 | 1.03 | 8.62 | 0.13 | 12.22 | 0.56 | 11.75 | --- | 12.15 | --- |
| North African Middle Pleistocene | --- | --- | --- | --- | 8.4 | 0.14 | 11.77 | 0.47 | 12.48 | 0.42 | 13.67 | 0.87 |
| East Asian Early Pleistocene | 11.06 | 0.45 | 8.21 | 0.15 | 7.95 | 0.58 | 10.85 | 0.89 | 12.00 | 0.74 | 13.01 | 0.68 |
| East Asian mid-Middle Pleistocene | 10.51 | 0.44 | 7.86 | 0.29 | 8.4 | 0.70 | 11.88 | 0.91 | 11.53 | 1.06 | 12.57 | 0.71 |
| East Asian Late Middle Pleistocene | --- | --- | --- | --- | 8.5 | 0.28 | 11.05 | 1.48 | 11.65 | --- | 13.50 | --- |
| East Asian Late Pleistocene | --- | --- | --- | --- | 6.35 | --- | 10.35 | --- | --- | --- | --- | --- |
| West Asian Early Pleistocene | 12.60 | --- | 7.8 | --- | 8.6 | --- | 11.6 | --- | 12.75 | 0.35 | 12.95 | 0.21 |
| West Asian Late Pleistocene | 10.11 | 0.87 | 8.23 | 0.73 | 7.77 | 0.55 | 10.45 | 0.37 | 11.63 | 0.64 | 12.34 | 0.72 |
| European Early Pleistocene | --- | --- | --- | --- | 8.4 | 0.36 | 11.63 | 0.15 | 12.03 | 0.11 | 12.58 | 0.75 |
| European Middle Pleistocene | 9.55 | 0.50 | 7.75 | 0.34 | 7.91 | 0.60 | 10.58 | 0.78 | 11.24 | 0.86 | 11.78 | 1.03 |
| Neanderthals | 9.46 | 1.00 | 8.54 | 0.64 | 7.81 | 0.71 | 10.60 | 0.69 | 11.49 | 0.92 | 12.09 | 0.79 |
| European Late Pleistocene (Early modern human) | 9.22 | 0.59 | 7.52 | 0.48 | 7.09 | 0.57 | 9.47 | 0.62 | 10.89 | 0.60 | 12.35 | 0.74 |
| Recent modern human | 9.26 | 0.68 | 7.47 | 0.39 | 6.95 | 0.43 | 9.52 | 0.53 | 10.45 | 0.49 | 12.05 | 0.50 |

**Table S5.** Mean and Std. Deviation of the crown size of the Hexian teeth and comparative samples (continued).

|  | M^2^ | | | | M_2_ | | | | M_3_ | | | |
| --- | --- | --- | --- | --- | --- | --- | --- | --- | --- | --- | --- | --- |
|  | MD | | BL | | MD | | BL | | MD | | BL | |
|  | Mean | SD | Mean | SD | Mean | SD | Mean | SD | Mean | SD | Mean | SD |
| Hexian | 12.25 | 0.35 | 14.75 | 1.06 | 13.95 | 0.49 | 13.65 | 0.35 | 13.30 | --- | 13.60 | --- |
| Pliocene (*Australopithecus*) | 13.79 | 1.63 | 15.63 | 1.80 | 15.16 | 1.39 | 14.18 | 1.19 | 15.99 | 1.46 | 14.43 | 1.31 |
| African Late Pliocene and Early Pleistocene (Early *Homo*) | 12.90 | 0.88 | 14.56 | 1.42 | 14.63 | 1.15 | 13.32 | 1.03 | 15.23 | 0.67 | 13.16 | 0.87 |
| African Late Pliocene and Early Pleistocene (*H. ergaster*) | 12.30 | 0.79 | 13.05 | 1.10 | 13.21 | 0.95 | 12.37 | 0.99 | 13.73 | 0.79 | 12.43 | 0.69 |
| North African Middle Pleistocene | 12.05 | 0.49 | 13.65 | 0.71 | 13.93 | 1.00 | 12.59 | 0.88 | 12.53 | 0.46 | 11.98 | 0.66 |
| East Asian Early Pleistocene | 12.17 | 1.01 | 13.40 | 1.11 | 13.03 | 1.74 | 12.33 | 1.63 | 13.34 | 1.53 | 12.09 | 1.19 |
| East Asian mid-Middle Pleistocene | 10.43 | 0.21 | 12.83 | 0.55 | 12.25 | 0.53 | 11.86 | 0.53 | 11.30 | 1.17 | 11.03 | 0.96 |
| East Asian Late Middle Pleistocene | 11.60 | --- | 14.1 | --- | --- | --- | --- | --- | --- | --- | --- | --- |
| East Asian Late Pleistocene | --- | --- | --- | --- | --- | --- | --- | --- | --- | --- | --- | --- |
| West Asian Early Pleistocene | 12.15 | 0.49 | 12.55 | 0.64 | 13.07 | 0.86 | 11.77 | 1.12 | 11.65 | 0.07 | 11.95 | 1.91 |
| West Asian Late Pleistocene | 10.91 | 1.10 | 12.31 | 0.72 | 11.40 | 0.71 | 11.00 | 0.91 | 11.86 | 0.96 | 10.70 | 0.70 |
| European Early Pleistocene | 12.10 | --- | 13.70 | --- | 12.47 | 0.47 | 12.10 | 1.42 | 10.65 | 2.05 | 9.60 | 1.13 |
| European Middle Pleistocene | 10.36 | 1.22 | 12.49 | 1.09 | 11.38 | 1.11 | 10.56 | 0.99 | 11.41 | 0.85 | 9.93 | 0.88 |
| Neanderthals | 10.82 | 0.96 | 12.61 | 0.72 | 11.99 | 0.82 | 11.25 | 0.64 | 11.69 | 0.68 | 10.95 | 0.96 |
| European Late Pleistocene (Early modern human) | 10.59 | 0.76 | 12.48 | 1.01 | 11.14 | 0.94 | 10.87 | 0.78 | 11.22 | 1.26 | 10.77 | 0.98 |
| Recent modern human | 9.74 | 0.64 | 11.85 | 0.75 | 10.66 | 0.63 | 10.49 | 0.50 | 10.43 | 0.66 | 10.28 | 0.59 |


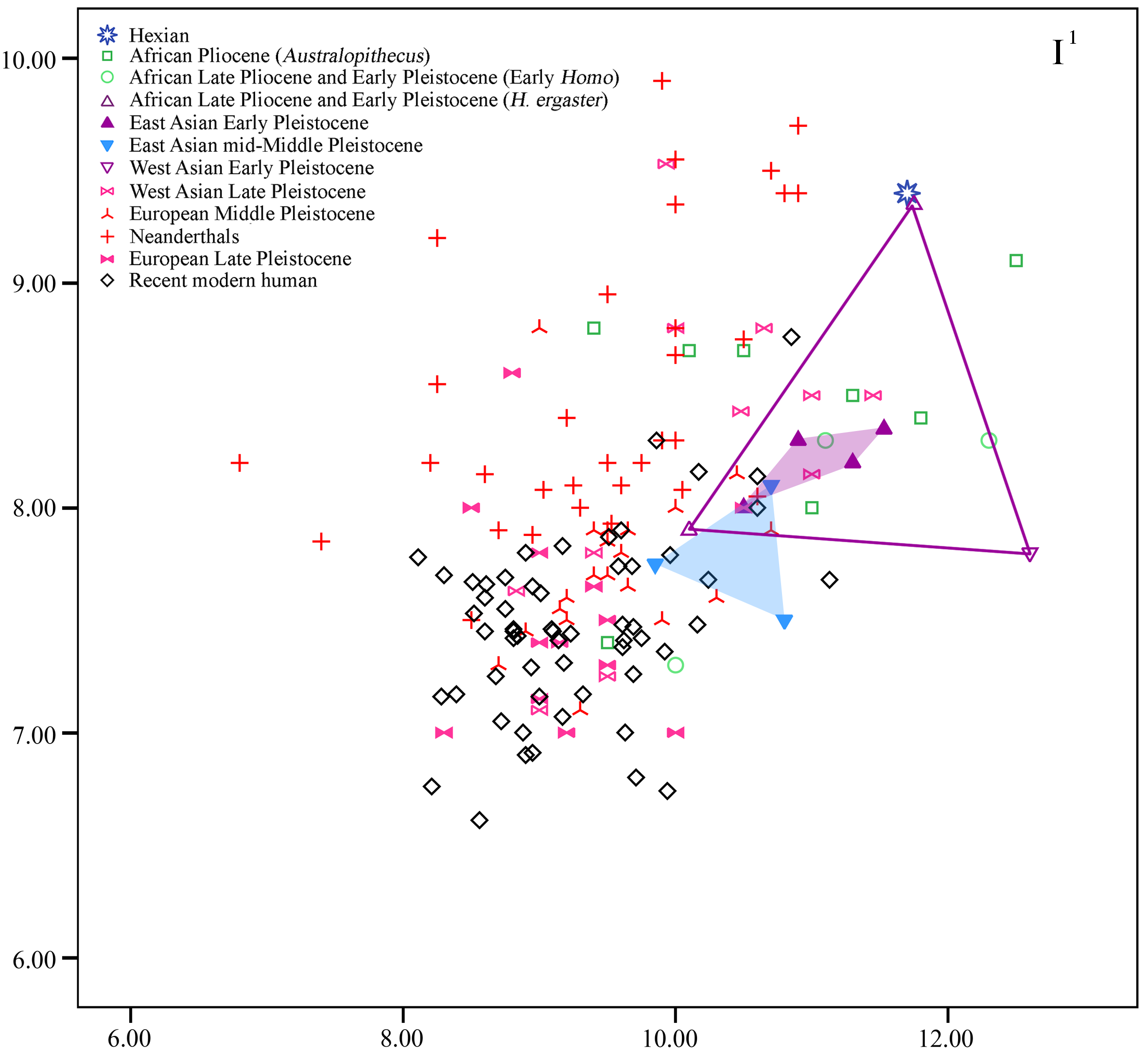


**Figure S1**. Bivariate plots of the crown sizes of I^1^s of the Hexian and the comparative samples (convex hulls were used to graphically highlight the distribution area of *H. erectus* sensu lato).


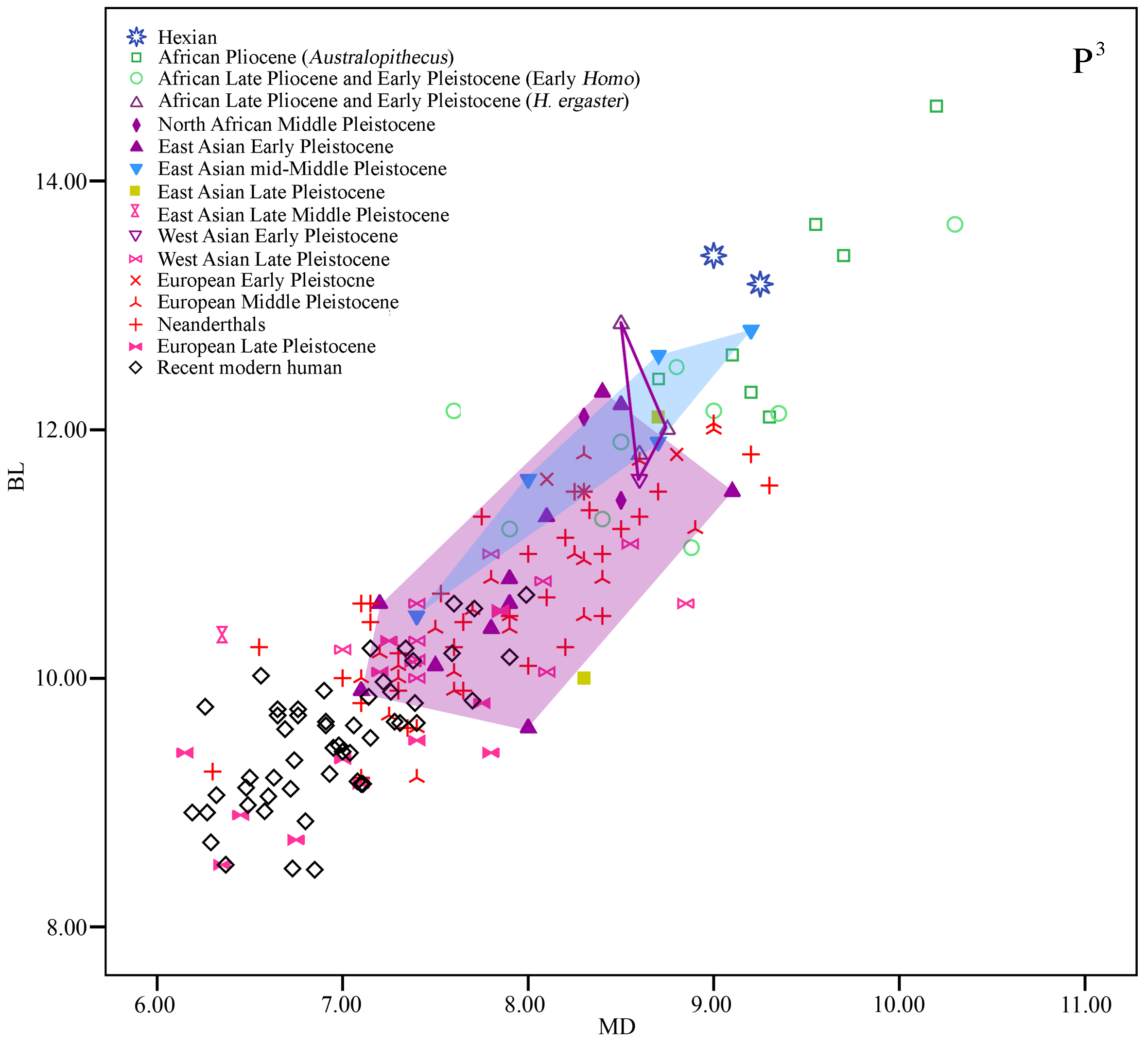


**Figure S2**. Bivariate plots of the crown sizes of P^3^s of the Hexian and the comparative samples (convex hulls were used to graphically highlight the distribution area of *H. erectus* sensu lato).


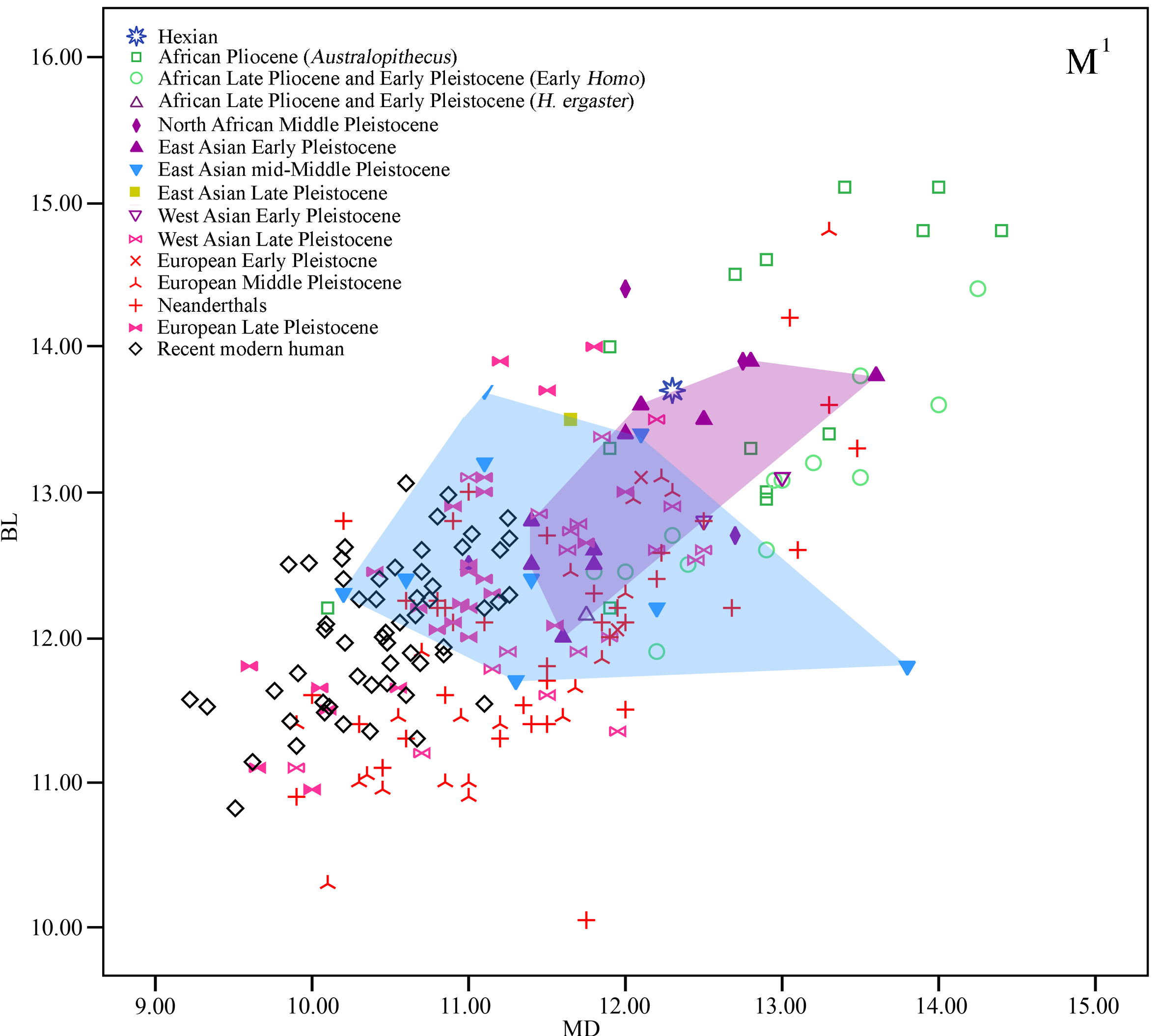


**Figure S3**. Bivariate plots of the crown sizes of M^1^s of the Hexian and the comparative samples (convex hulls were used to graphically highlight the distribution area of *H. erectus* sensu lato).


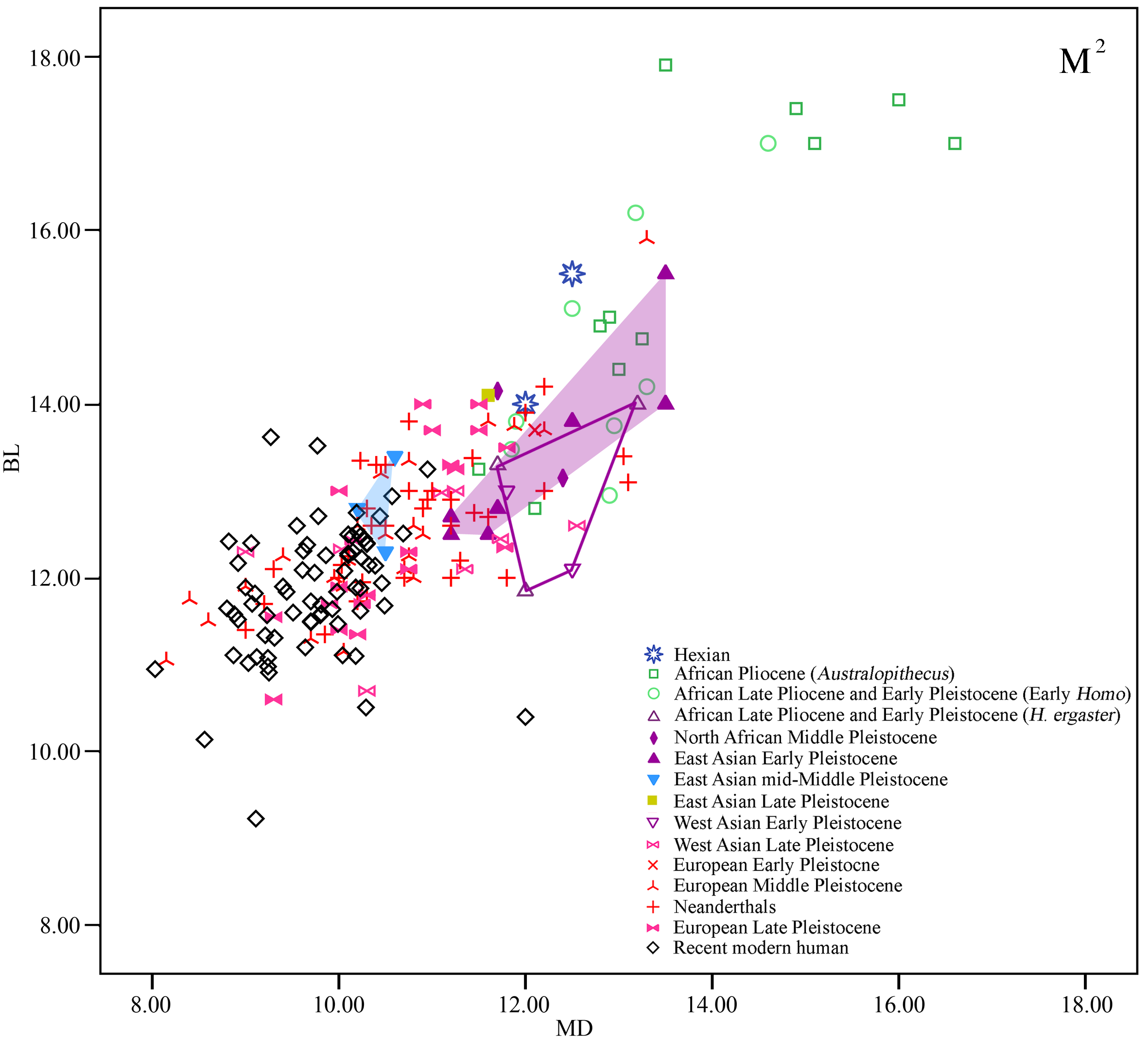


**Figure S4**. Bivariate plots of the crown sizes of M^2^s of the Hexian and the comparative samples (convex hulls were used to graphically highlight the distribution area of *H. erectus* sensu lato).


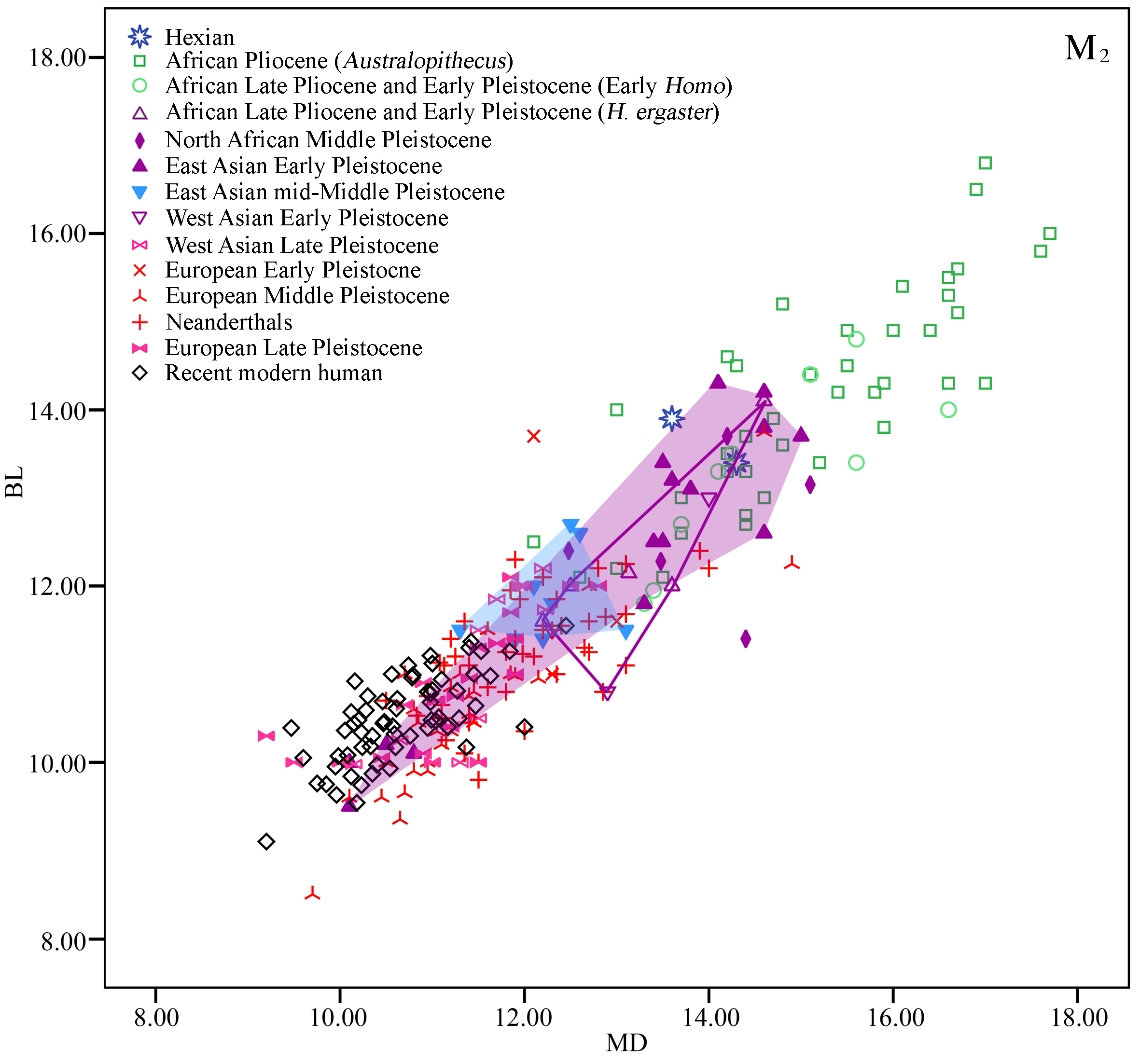


**Figure S5**. Bivariate plots of the crown sizes of M_2_s of the Hexian and the comparative samples (convex hulls were used to graphically highlight the distribution area of *H. erectus* sensu lato).


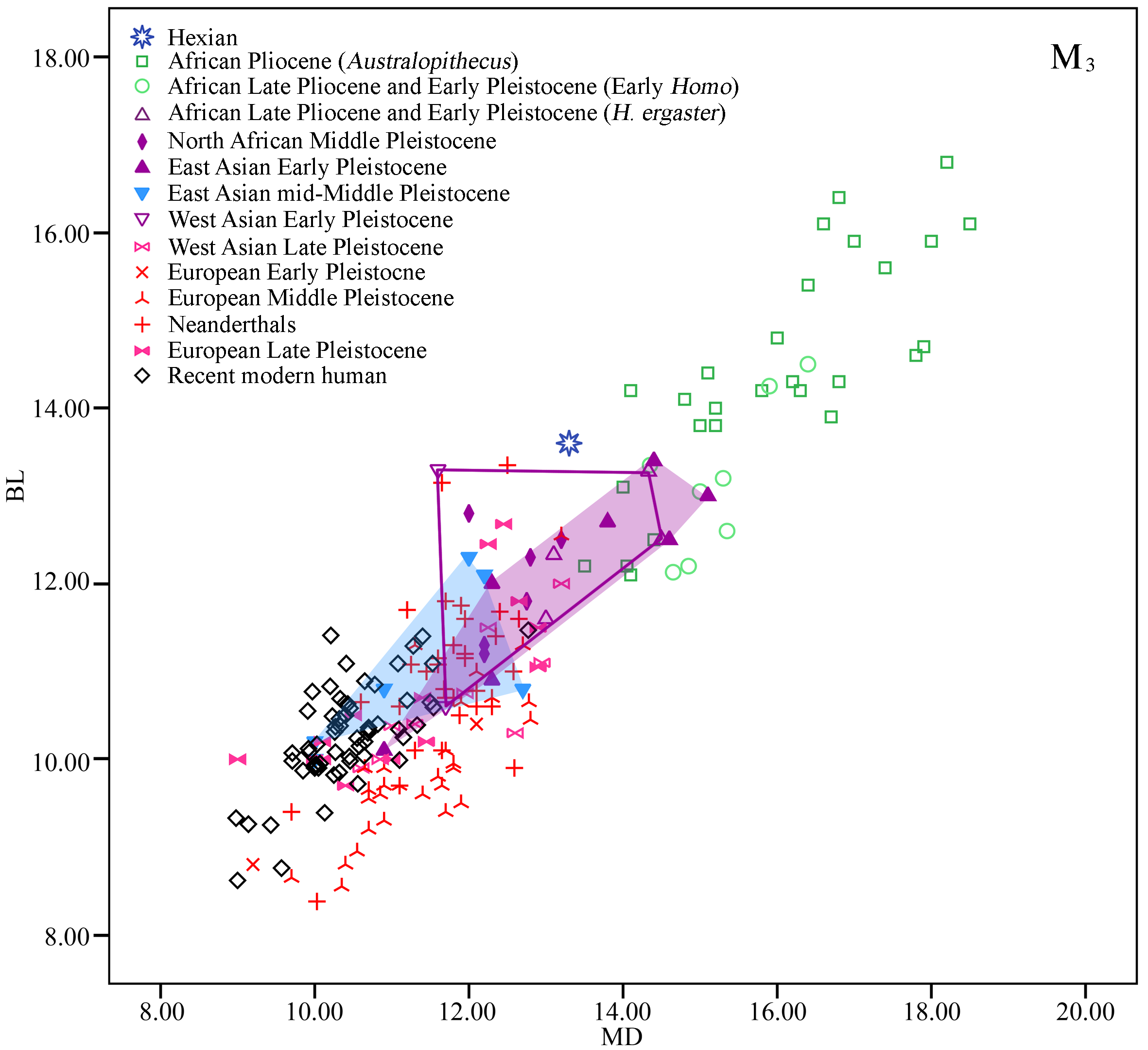


**Figure S6**. Bivariate plots of the crown sizes of M_3_s of the Hexian and the comparative samples (convex hulls were used to graphically highlight the distribution area of *H. erectus* sensu lato).

**SI References**

1. Huang W, Fang D, Ye Y (1982) Preliminary study on the fossil hominid skull and fauna of Hexian, Anhui. Vertebrate PalAsiatica 20: 248-257.
2. Zheng S (1983) The Middle Pleistocene micromammalian fauna from Hexian man locality and its significance. Chin Sci Bull 28: 237-239.
3. Li H, Mei Y (1983) The maximum age of Hexian man. Chin Sci Bull 28: 1146-1147.
4. Chen T, Yuan S, Gao S, Hu Y (1987) Uranium series dating of fossil bones from Hexian and Chaoxian fossil human sites. Acta Anthropol Sinica 6: 249-254.
5. Huang P, Zheng L, Quan Y, Liang R, Xu Y, et al. (1994) Preliminary study on ESR method for dating age of Hexian man. Chin Sci Bull 40: 1318-1320.
6. Huang P, Liang R, Zheng L, Quan Y, Xu Y, et al. (1995a) Study on the Hexian human fossil age. Acta Anthropol Sinica 14: 262-265.
7. Huang P, Zheng L, Quan Y (1995b) Preliminary study on ESR dating of Hexian-Man and its fauna. Nucl Tech 18: 491-494.
8. Grün R, Huang P, Huang W, Dermott FM, Thorne A, et al. (1998) ESR and U-series analyses of teeth from the palaeoanthropological site of Hexian, Anhui Province, China. J Hum Evol 34: 555-564.
9. Moggi-Cecchi J, Grine FE, Tobias PV (2006) Early hominid dental remains from Members 4 and 5 of the Sterkfontein Formation (1966-1996 excavations): Catalogue, individual associations, morphological descriptions and initial metrical analysis. J Hum Evol 50: 239-328.
10. Tobias PV (1991) Olduvai Gorge. In: The Skulls, Endocasts and Teeth of *Homo habilis*, volume 4. Cambridge:Cambridge University Press
11. Wood BA (1991) Koobi Fora Research Project, vol. 4: Hominid Cranial Remains. Oxford: Clarendon Press.
12. Walker A, Leakey RE (1993) The Nariokotome *Homo erectus* skeleton. Cambridge, MA: Harvard University Press.
13. Arambourg C, Biberson P (1956) The fossil human remains from the paleolithic site of Sidi Abderrahman (Morocco). Am J Phys Anthropol 14: 467-489.
14. Tobias PV, von Koenigswald GHR (1964) A comparison between the Olduvai hominines and those of Java and some implications for hominid phylogeny. Nature 204: 515–518.
15. Hu C (1973) Ape-man teeth from Yuanmou, Yunnan, Acta Geol Sinica 1: 65-71.
16. Jacob T (1973) Palaeoanthropological Discoveries in Indonesia With Special Reference to the Finds Department of Physical of the Last Two Decades. J Hum Evol 2: 473-485.
17. Grine FE, Franzen JL (1994) Fossil hominid teeth from the Sangiran Dome (Java, Indonesia). Cour Forschungsinst Senckenb, 171: 75-103.
18. Arif J, Kaifu Y, Baba H, Suparka M E, Zaim Y et al. (2002) Preliminary Observation of a New Cranium of *Homo erectus* (Tjg-1993.05) from Sangiran, Central Jawa. Anthropol Sci 110: 165-177.
19. Tyler DE (2004) An examination of the taxonomic status of the fragmentary mandible Sangiran 5, (*Pithecanthropus dubius*), *Homo erectus*, “*Meganthropus*”, or *Pongo*? Quatern Int 117, 125-130.
20. Kaifu Y, Aziz F, Baba H (2005) Hominid mandibular remains from Sangiran: 1952–1986 collection. Am J Phys Anthropol 128: 497-519.
21. Zaim Y, Ciochon RL, Polanski JM, Grine FE, Bettis EA et al. (2011) New 1.5 million-year-old *Homo erectus* maxilla from Sangiran (Central Java, Indonesia). J Hum Evol 61: 363-376.
22. Zanolli C (2013) Additional Evidence for Morpho-Dimensional Tooth Crown Variation in a New Indonesian *H. erectus* Sample from the Sangiran Dome (Central Java). PLoS ONE 8(7): e67233. doi: 10.1371/journal.pone.0067233.
23. Weidenreich F (1937) The Dentition of *Sinanthropus Pekinensis*: A Comparative Odontography of the Hominids. Palaeontologica Sinica N.S. D1. The Geological Survey of China.
24. Woo J, Chia L (1954) New discoveries of *Sinanthropus pekinensis* in Choukoutien. Acta Palaeontologica Sinica 2: 267-288.
25. Bailey SE, Liu W (2010) A comparative dental metrical and morphological analysis of a Middle Pleistocene hominin maxilla from Chaoxian (Chaohu), China. Quatern Int 211: 14–23.
26. Liu W, Schepartz LA, Xing S, Miller-Antonio S, Wu X et al. (2013) Late Middle Pleistocene hominin teeth from Panxian Dadong, South China, J Hum Evol 64: 337-355.
27. Martinón-Torres M, Bermúdez de Castro JM, Gómez-Robles A, Margvelashvili A, Prado L, et al. (2008) Dental remains from Dmanisi (Republic of Georgia): morphological analysis and comparative study. J Hum Evol 55: 249-273.
28. Bermúdez de Castro JM, Rosas A, Nicolás ME (1999) Dental remains from Atapuerca-TD6 (Gran Dolina site, Burgos, Spain). J Hum Evol 37: 523–566.
29. Carbonell E, Bermúdez de Castro JM, Arsuaga JL, Allue E, Bastir M et al. (2005) A new early Pleistocene hominin mandible from Atapuerca-TD6, Spain. Proc Natl Acad Sci USA 102: 5674-5678.
30. Martinón-Torres M, Bermúdez de Castro JM, Gómez-Robles A, Prado-Simón L, Arsuaga JL (2012) Morphological description and comparison of the dental remains from Atapuerca-Sima de los Huesos site (Spain). J Hum Evol 62: 7-58.
31. Frayer DW (1977) Metric dental change in the European upper paleolithic and Mesolithic. Am J Phys Anthropol 46: 109–120.
32. Turner CG II, Nichol CR, Scott GR (1991) Scoring procedures for key morphological traits of the permanent dentition: the Arizona State University dental anthropology system. In: Kelley M, Larsen C, editors. Advances in Dental Anthropology. New York: Wiley-Liss. 13-31 p.
